# Ménage-à-Trois: The Ratio of Bicarbonate to CO_2_ and the pH Regulate the Capacity of Neutrophils to Form NETs

**DOI:** 10.3389/fimmu.2016.00583

**Published:** 2016-12-09

**Authors:** Christian Maueröder, Aparna Mahajan, Susanne Paulus, Stefanie Gößwein, Jonas Hahn, Deborah Kienhöfer, Mona H. Biermann, Philipp Tripal, Ralf P. Friedrich, Luis E. Munoz, Markus F. Neurath, Christoph Becker, Georg Andreas Schett, Martin Herrmann, Moritz Leppkes

**Affiliations:** ^1^Department of Internal Medicine 3 – Rheumatology and Immunology, Universitätsklinikum Erlangen, Friedrich-Alexander-University Erlangen-Nürnberg (FAU), Erlangen, Germany; ^2^Department of Medicine 1 – Gastroenterology, Pulmonology and Endocrinology, Universitätsklinikum Erlangen, Friedrich-Alexander-University Erlangen-Nürnberg (FAU), Erlangen, Germany; ^3^Optical Imaging Center Erlangen (OICE), Friedrich-Alexander-University Erlangen-Nürnberg (FAU), Erlangen, Germany; ^4^ENT Clinic, Section of Experimental Oncology and Nanomedicine (SEON), University Hospital Erlangen, Friedrich-Alexander-University Erlangen-Nürnberg (FAU), Erlangen, Germany

**Keywords:** NET, pH, inflammation, bicarbonate, CO_2_, neutrophils, neutrophil extracellular traps, calcium

## Abstract

In this study, we identified and characterized the potential of a high ratio of bicarbonate to CO_2_ and a moderately alkaline pH to render neutrophils prone to undergo neutrophil extracellular trap (NET) formation. Both experimental settings increased the rate of spontaneous NET release and potentiated the NET-inducing capacity of phorbol esters (phorbol-2-myristate-13-acetate), ionomycin, monosodium urate, and LPS. In contrast, an acidic environment impaired NET formation both spontaneous and induced. Our findings indicate that intracellular alkalinization of neutrophils in response to an alkaline environment leads to an increase of intracellular calcium and neutrophil activation. We further found that the anion channel blocker DIDS strongly reduced NET formation induced by bicarbonate. This finding suggests that the effects observed are due to a molecular program that renders neutrophils susceptible to NET formation. Inflammatory foci may be characterized by an acidic environment. Our data indicate that NET formation is favored by the higher pH at the border regions of inflamed areas. Moreover, our findings highlight the necessity for strict pH control during assays of NET formation.

## Introduction

Neutrophils are the most abundant leukocyte subset in the human blood and constitute the first line of defense during infection (1, 2). A central effector function of neutrophils involves the release of decondensed chromatin decorated with cytoplasmic and granular proteins (3, 4). Since these structures may trap and degrade pathogens extracellularly inside their meshwork, they are referred to as neutrophil extracellular traps (NETs) and the accompanying process is termed NET formation (3). A variety of stimuli have been reported to induce formation of NETs, among them are bacteria, fungi, and microbial products (3, 5, 6). Other physiological stimuli include monosodium urate, immune complexes, apoptotic cells, or integrin-mediated signals at high cellular density (7–10). In experimental settings, chemicals with defined mechanisms of action such as phorbol-2-myristate-13-acetate (PMA) or ionomycin also induce NET formation (4). We have recently reported that occlusion of the pancreatic ducts by aggregated NETs is a driving factor of pancreatitis (11). In this study, we identified bicarbonate present in the pancreatic juice as a potent inducer of NET formation. In this manuscript, we aim to characterize more closely the influence of the triangular relationship of bicarbonate, CO_2_, and pH on NET formation. We observed that both a low pH and a high CO_2_ to bicarbonate ratio decrease the capacity of neutrophils to release NETs. Inflammatory foci may be characterized by an acidic microenvironment. Our data indicate that NET formation is favored at the border regions of inflamed areas and the beginning of inflammation. Furthermore, NET release may be favored by the restitution of physiological pH in ischemia–reperfusion situations. These observations will impact the understanding of multiple inflammatory diseases.

## Materials and Methods

### Chemicals

4,4′-Diisothiocyanatostilbene-2,2′-disulfonic acid disodium salt hydrate (DIDS) and PI were from Sigma (Crailsheim, Germany). Hoechst 33342 was obtained from Thermo Fisher Scientific (Frankfurt, Germany).

### Isolation of Polymorphonuclear Leukocytes

All analyses of human material were performed in accordance to the institutional guidelines and with the approval of the ethical committee of the University Hospital Erlangen (permit # 193 13B). Written informed consent was given by each donor. Twenty milliliter of heparinized blood (20 U/ml) were taken from each normal healthy donor. Fifteen milliliter of PBS without calcium and magnesium (Thermo Fisher Scientific) were added and the suspension was gently applied on top of 15 ml of Ficoll (Bio-Rad, Dreieich, Germany). Cells were centrifuged at 1,400 rpm for 30 min at room temperature with lowest acceleration. Centrifuge was allowed to spin out without break. Suspension above the buffy coat was removed, and the white layer containing the PMNs on the top of the red blood cells was collected. To remove contaminating erythrocytes, PMNs were subjected to short cycles of hypotonic lysis with deionized water. Normal osmolality was restituted after 30 s with 10× PBS. PMNs with purity higher than 95% were adjusted to a concentration of 2 × 10^6^ cells/ml in PBS without calcium and magnesium (Thermo Fisher Scientific) and stored at room temperature until further use.

### Buffers and Culture Conditions

Isolated neutrophils were adjusted to a concentration of 6 × 10^6^ cells/ml in Ringer’s solution (Deltaselect, Pfullingen, Germany). Twenty-five microliter of these solutions were added to 175 μl of indicated medium containing 2.5 μM Sytox Green (Thermo Fisher Scientific) with or without 10 ng/ml PMA (Sigma, Darmstadt, Germany), 1 μg/ml ionomycin (Sigma), 300 pg/cell monosodium urate crystals, or 2.5 μg/ml LPS from *Klebsiella pneumoniae* (L4268, Sigma) or *Salmonella enterica* serotype enteritidis (L6143, Sigma), respectively. Assays were performed either in 96-well cell plates (Greiner Bio-One, Frickenhausen, Germany) or 8-well Nunc chamber slides (VWR, Darmstadt, Germany). Plates and chamber slides were preincubated at 37°C and respective concentrations of CO_2_ at least 30 min prior to addition of 25 μl of cells in Ringer.

### Platereader-Based Quantification of NET Formation

Plates containing PMN cultures were analyzed under the conditions described above for 4 h on an infinite^®^ 200 pro plate reader (TECAN, Crailsheim, Germany). Excitation was performed at 485 nm and emission was detected at 535 nm. Relative fluorescence units were calculated as the 100-fold ratio of the fluorescence at the indicated time point and time point *t* = 0 min.

### Immunohistochemistry

After addition of the cells, the chamber slides were incubated under these conditions for 3 h. Subsequently, 1% paraformaldehyde (Merck, Darmstadt, Germany) in PBS (Thermo Fisher Scientific) was added to each well and the preparations were incubated for 18 h at 4°C. Samples were blocked with 10% FCS (Biochrome, Berlin, Germany) in PBS (Thermo Fisher Scientific) for 1 h at room temperature. Cells were permeabilized with 0.1% Triton X-100 in water for 10 min. Primary antibody for neutrophil elastase (NE) (Abcam, United Kingdom, ab21595) 1:200 or citrullinated histone H3 (citH3) (Abcam, ab5103) 1:200 were added in 10% FCS in PBS for 18 h at 4°C. Slides were washed three times with PBS, and secondary anti-rabbit IgG antibody conjugated with Cy^®^5 (Jackson ImmunoResearch, Suffolk, United Kingdom, 111-175-144) 1:400 was added for 1.5 h at room temperature in the dark. Slides were washed with PBS. Staining solution containing 2.5 μM Sytox Green in PBS was added for 15 min at room temperature. Slides were washed with H_2_O and samples were embedded in DAKO fluorescent mounting medium (BIOZOL, Eching, Germany). Slides were analyzed on a BZ-X710 microscope (Keyence, Neu-Isenburg, Germany). Maximum intensity projection of Z-stacks and gamma correction were performed to increase depth of field and to allow proper display of NETs and nuclei on these images. Post-processing of pictures was performed with Photoshop CS5 (Adobe, München, Germany). Images were not used for quantification.

### Live Cell Imaging

Chamber slides containing PMN cultures under the conditions described above were analyzed on a BZ-X710 microscope (Keyence, Neu-Isenburg, Germany) or an Axio Observer.Z1 microscope (Zeiss, Oberkochen, Germany) using a time-lapsed shooting sequence. Maximum intensity projection of Z-stacks and gamma correction were performed to increase depth of field and to allow proper display of NETs and nuclei on the same image, respectively. Post-processing of pictures was performed with Photoshop CS5 (Adobe, München, Germany) and ZEN pro 2012 (Zeiss).

### Intracellular Calcium Measurement

Isolated neutrophils were suspended at a concentration of 10 × 10^6^ cells/ml in PBS without calcium and magnesium and loaded with 3 μM Fluo-3 AM (Thermo Fisher Scientific) and 6 μM Fura-red AM (Thermo Fisher Scientific). Cells were incubated for 20 min at room temperature and followed by incubation at 37°C for 10 min. Cells were washed twice with PBS without calcium and magnesium and suspended in same medium at final concentration of 10 × 10^6^ cells/ml. Fifty microliter of cell suspension was added to 450 μl of PBS with 0.4 mM CaCl_2_ and measured for 1 min by flow cytometry. Then, 2.5 ml of respective medium with calcium was added, which was preincubated at 37°C and 5% CO_2_ and fluorescence was measured for 15 min. To study the effect of extracellular acidification/alkalinization on calcium mobilization, PMN loaded with Fluo-3 AM and Fura-red AM was measured by flow cytometry in respective preincubated medium for 1 min. Then predetermined amount of HCl or NaOH was added to achieve respective extracellular pH followed by measurement for 15 min. Beckman Coulter’s Epics XL-MCL™ and software Kaluza 1.5 (Beckmann Coulter) were used for measurement and analysis, respectively. Original data file of cell events was divided in time-based gates and ratiometric fluorescence FL1–FL3 was used to determine intracellular calcium levels. The radiometric calcium levels were normalized to first time point level.

### Measurement of Intracellular pH

Isolated neutrophils (10 × 10^6^ cells/ml in PBS without calcium and magnesium) were loaded with 10 μM carboxy-SNARF-1-AM (Thermo Fisher Scientific) and incubated at room temperature for 20 min followed by incubation at 37°C for 10 min. Cells were washed twice with PBS without calcium and magnesium and suspended in same buffer at 10 × 10^6^ cells/ml. Intracellular pH of neutrophils with different concentrations of bicarbonate in RPMI was recorded using Gallios Flow Cytometer (Beckman Coulter, USA). To determine the change in intracellular pH in response to extracellular pH of the medium, 100 μl of the cell suspension was added to 2.9 ml of respective medium preincubated at 37°C and 5% CO_2_ and fluorescence was recorded for 1 min using Gallios Flow Cytometer (Beckman Coulter, USA) and respective extracellular pH was attained using predetermined volume of HCl and NaOH followed by flow cytometry measurement for 15 min. The change in intracellular pH was determined by ratio of FL6–FL2 in Beckman Coulter analysis software Kaluza 1.5.

### Data Presentation and Statistical Analysis

Results are displayed as means ± SEM of the indicated number of biological replicates. If not indicated otherwise, an analysis of variance was used for statistical analysis. In case of multiple comparisons, Tukey’s correction was performed. Statistical significance is indicated with *, **, *** and ****. The respective confidential intervals are *p* < 0.05, *p* < 0.01, *p* < 0.001, and *p* < 0.0001. Statistical analysis was performed with the software GraphPad Prism 6.0 (GraphPad Software, USA).

## Results

**Figure 1:**
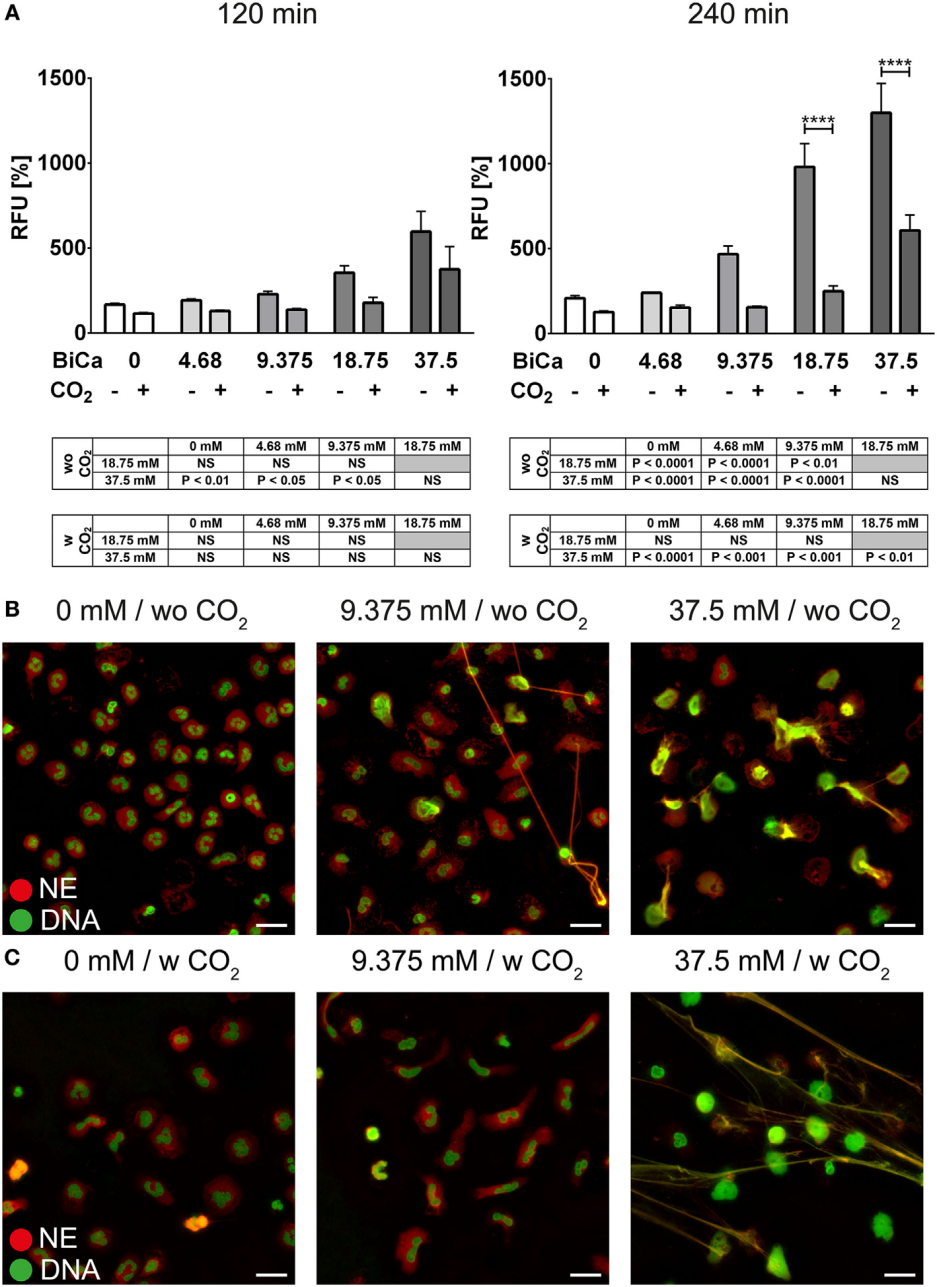
**Bicarbonate induces formation of neutrophil extracellular traps in HBSS**. HBSS was supplemented with various concentrations of bicarbonate either in the absence (−) or presence (+) of 5% CO_2_. **(A)** Fluorescence-based quantification of DNA externalization in response to various concentrations of bicarbonate after 120 min (left) and 240 min (right). *n* = 3–5. **(B)** Immunocytochemical analysis of neutrophils incubated in the presence of various concentrations of bicarbonate. Signal for DNA is depicted in green, and signal for neutrophil elastase (NE) is displayed in red. The scale bar represents 20 μm. **(C)** Immunocytochemical analysis of neutrophils incubated in the presence of various concentrations of bicarbonate in the presence of 5% CO_2_. Signal for DNA is depicted in green, and signal for NE is displayed in red. The scale bar represents 20 μm.

Freshly isolated PMNs were cultured in HBSS containing various amounts of bicarbonate. Quantification of DNA release in a fluorescence-based assay revealed that bicarbonate time- and dose-dependently induces an increase in Sytox Green signal (Figure 1A). Immunocytochemistry revealed that increasing amounts of bicarbonate induced the formation of thread-like DNA structures positive for NE (Figure 1B; Figure S1 in Supplementary Material). Live cell imaging further revealed that excessive bicarbonate induces chromatin externalization from neutrophils (Video S1 in Supplementary Material). Together, these data indicate that bicarbonate is a potent determinant of whether culture media induce NET formation. Since the ratio of bicarbonate to CO_2_ influences the pH of the medium, we analyzed the impact of the extracellular pH on formation of NETs. Therefore, the medium was supplemented with 5% CO_2_. Indeed, we observed an ameliorative effect of CO_2_ supplementation, indicating an important role of the pH in bicarbonate-induced NET release (Figure 1C; Figure S2 in Supplementary Material).

**Figure 2:**
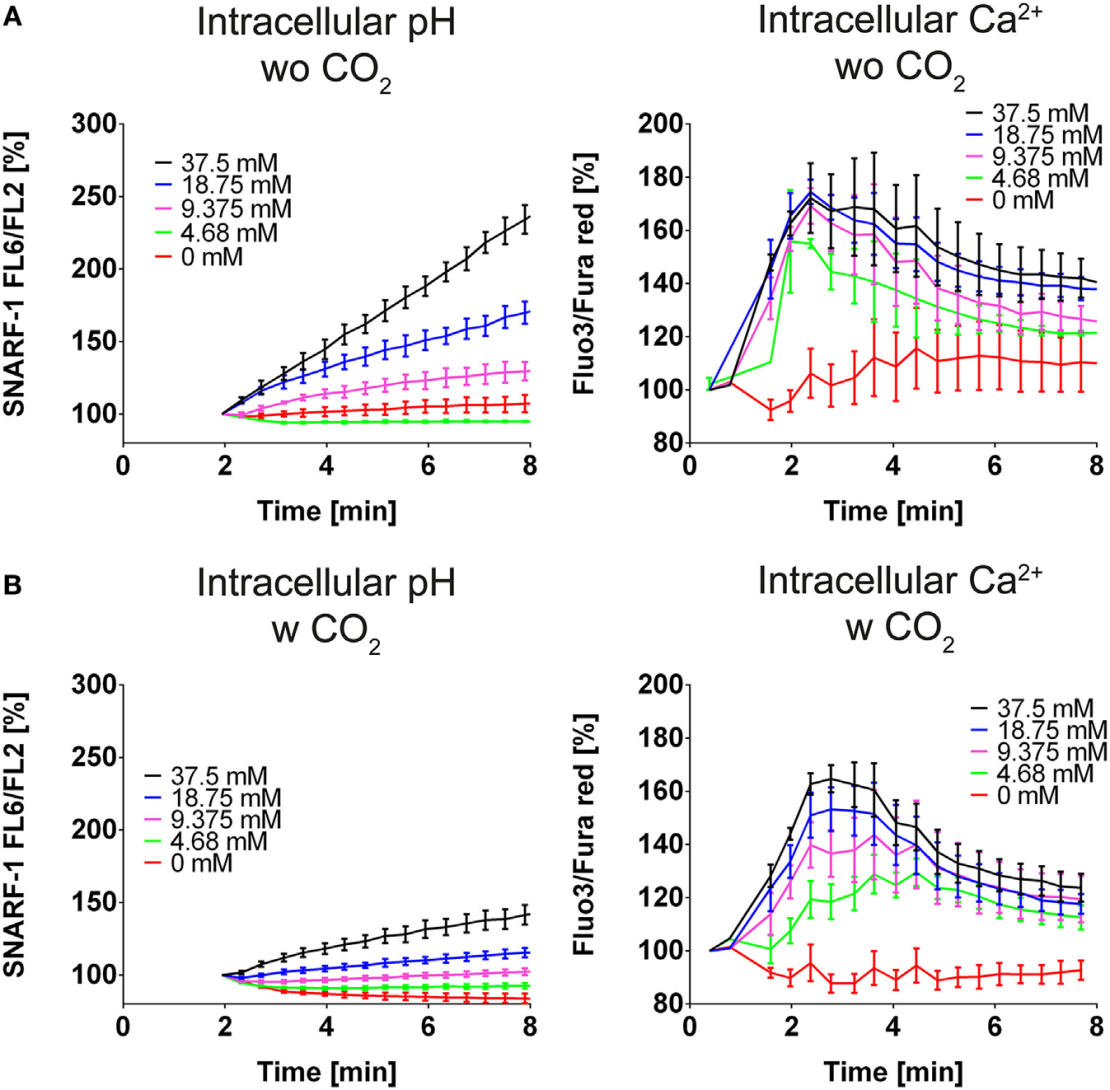
**A high ratio of bicarbonate to CO_2_ induces cytosolic alkalinization and intracellular calcium increase in HBSS**. HBSS was supplemented with various concentrations of bicarbonate either in the absence **(A)** or presence **(B)** of 5% CO_2_. **(A)** Flow cytometric determination of intracellular pH (left) and cytosolic Ca^2+^ concentration (right) of neutrophils at various concentrations of bicarbonate. *n* = 3. **(B)** Flow cytometric determination of intracellular pH (left) and cytosolic Ca^2+^ concentration (right) of neutrophils at various concentrations of bicarbonate. *n* = 3.

Many processes during NET formation, such as citrullination of histone H3, are dependent on calcium. Elevated levels of calcium are likely to render neutrophils more prone to release NETs. Intracellular alkalinization of neutrophils is reportedly accompanied by intracellular calcium increase. We observed that bicarbonate dose-dependently induces intracellular alkalinization and intracellular increase of calcium (Figure 2A). These effects were reduced when HBSS was supplemented with CO_2_, further highlighting the importance of the pH in bicarbonate-induced formation of NETs (Figure 2B).

**Figure 3:**
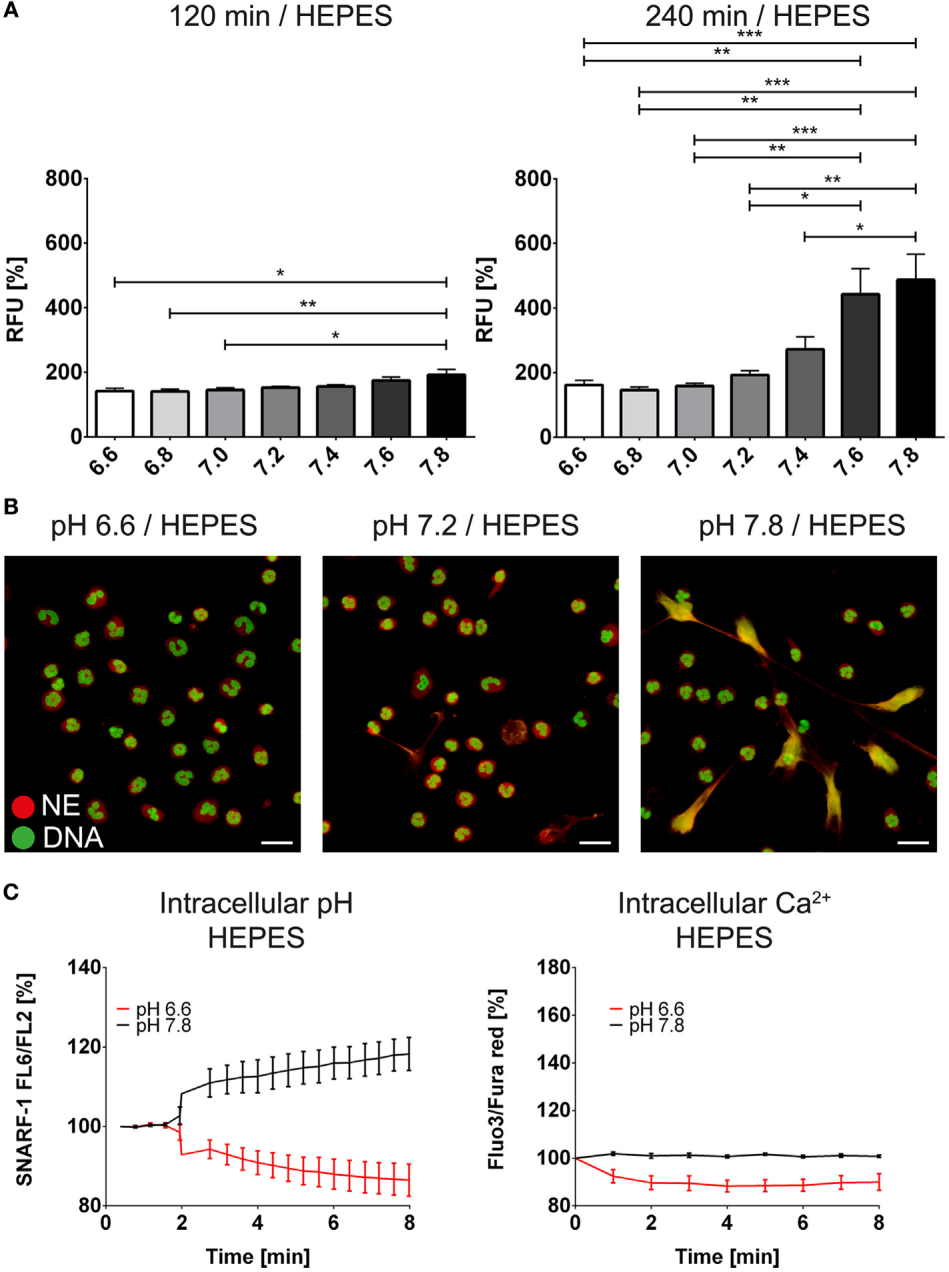
**High extracellular pH values induce formation of neutrophil extracellular traps in the absence of bicarbonate/CO_2_ in RPMI**. RPMI was supplemented with 20 mM HEPES, and the pH was adjusted to values reaching from 6.6 to 7.8 with hydrochloric acid and sodium hydroxide, respectively. **(A)** Fluorescence-based quantification of DNA externalization in response to various extracellular pH values after 120 min (left) and 240 min (right). **(B)** Immunocytochemical analysis of neutrophils incubated at a medium pH of 6.6, 7.2, and 7.8, respectively. Signal for DNA is depicted in green, and signal for neutrophil elastase is displayed in red. The scale bar represents 20 μm. **(C)** Flow cytometric determination of intracellular pH (left) and cytosolic Ca^2+^ concentration (right) of neutrophils at pH values of 6.6 and 7.8. *n* = 3–4.

We next analyzed whether pH-dependent effects are also observed independently of the bicarbonate/CO_2_ axis. Therefore, we deployed RPMI medium buffered with 20 mM HEPES. We tested a variety of pH values ranging from pH 6.6 to 7.8 and observed that with increasing alkalinity of the medium, more NET formation was observed as identified by immunocytochemistry and live cell imaging displaying the typical morphological characteristics of NETs (Figures 3A,B; Figure S3 in Supplementary Material; Video S2 in Supplementary Material). Importantly, Sytox Green binding to DNA was not influenced by the pH (Figure S4 in Supplementary Material). We further tested whether intracellular alkalinization and increase in Ca^2+^ concentration occur in the absence of bicarbonate and CO_2_. *Via* addition of hydrochloric acid and sodium hydroxide, the extracellular pH was adjusted to 6 and 7.8, respectively. Strikingly, such a manipulation was ineffective in influencing the intracellular pH (Figure 3C). In line with this, also the intracellular calcium concentration remained stable after acidification or alkalinization (Figure 3C).

So far, we had identified the bicarbonate/CO_2_ axis and the extracellular proton concentration as an important determinant of neutrophil behavior. These data highlight the necessity of strict CO_2_ control in media containing bicarbonate (i.e., HBSS and RPMI). Per example, cultivation of neutrophils in regular RPMI containing 24 mM bicarbonate for 30 min in the absence of proper CO_2_ control, induces robust formation of NET-like structures, which are not present if 5% CO_2_ is supplied (Figure S5 in Supplementary Material).

**Figure 4:**
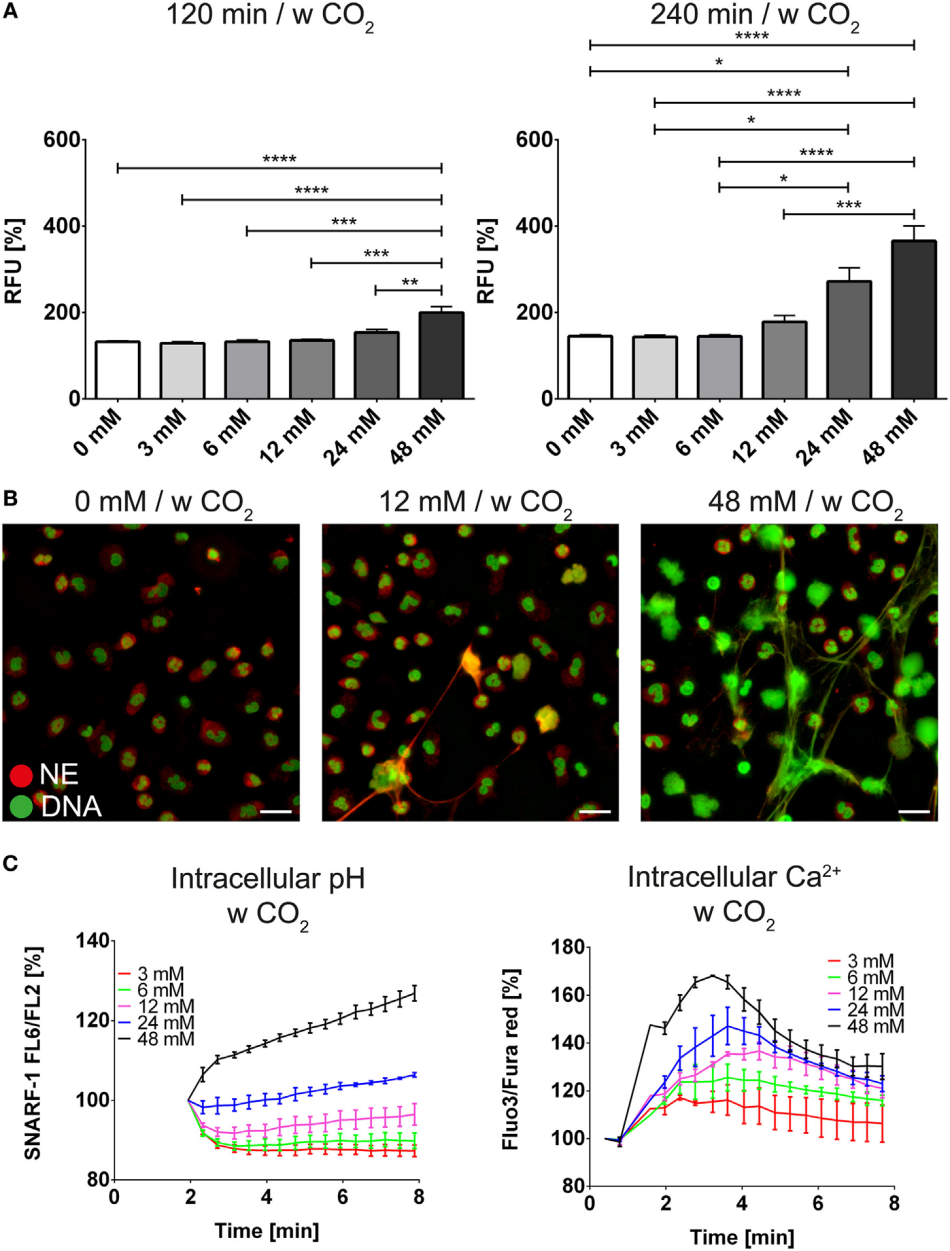
**The bicarbonate to CO_2_ ratio influences formation of neutrophil extracellular traps in RPMI**. RPMI was supplemented with various concentrations of bicarbonate in the presence of 5% CO_2_. **(A)** Fluorescence-based quantification of DNA externalization in response to various concentrations of bicarbonate after 120 min (left) and 240 min (right). *n* = 5–11. **(B)** Immunocytochemical analysis of neutrophils incubated in the presence of various concentrations of bicarbonate. Signal for DNA is depicted in green, and signal for neutrophil elastase is displayed in red. The scale bar represents 20 μm. **(C)** Flow cytometric determination of intracellular pH (left) and cytosolic Ca^2+^ concentration (right) of neutrophils at various concentrations of bicarbonate. *n* = 3.

We wondered whether the extracellular pH was also able to modify the response of neutrophils toward known inducers of NET release such as PMA and ionomycin. In order to study the modulation of the intracellular pH by the bicarbonate/CO_2_ axis under more physiologic conditions, we deployed RPMI buffered with various concentrations of bicarbonate under 5% CO_2_ atmosphere. NET release in the absence of added inducers was increased at higher concentrations of bicarbonate (Figures 4A,B; Figure S6 in Supplementary Material; Video S3 in Supplementary Material) and we observed dose-dependent alkalinization and intracellular calcium increase (Figure 4C). Importantly, sustained elevation of intracellular calcium was only observed after intracellular alkalinization, whereas a mild and transient increase was also observed in response to acidification (Figure 4C).

**Figure 5:**
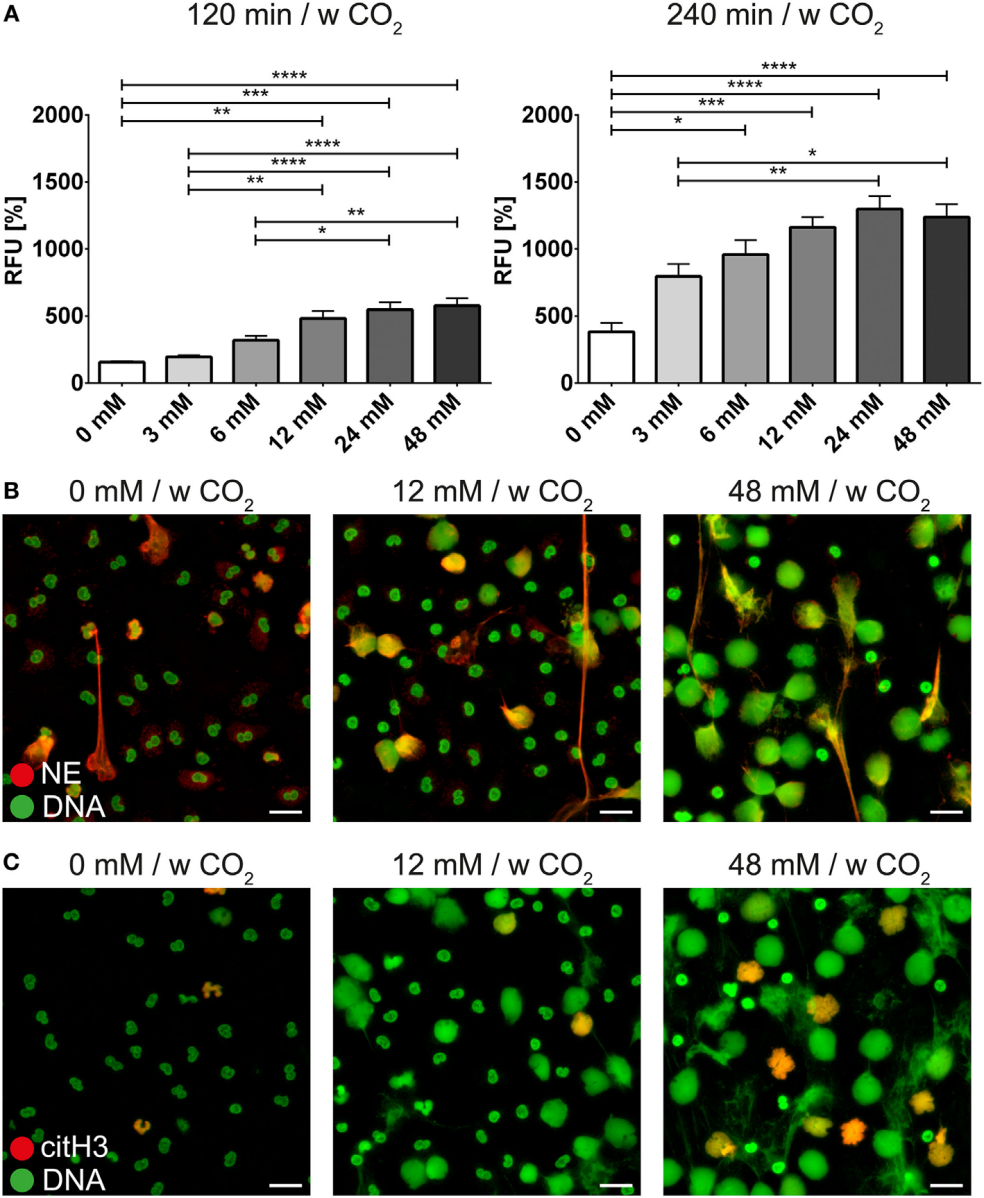
**The bicarbonate to CO_2_ ratio influences the neutrophil extracellular trap-inducing potential of phorbol-2-myristate-13-acetate (PMA) in RPMI**. RPMI was supplemented with various concentrations of bicarbonate in the presence of 5% CO_2_. PMA was used at a concentration of 10 ng/ml. **(A)** Fluorescence-based quantification of DNA externalization in response to various concentrations of bicarbonate after 120 min (left) and 240 min (right) in the presence of PMA. *n* = 5–11. **(B)** Immunocytochemical analysis of neutrophils incubated in the presence of various concentrations of bicarbonate in the copresence of PMA. Signal for DNA is depicted in green, and signal for neutrophil elastase is displayed in red. The scale bar represents 20 μm. **(C)** Immunocytochemical analysis of neutrophils incubated in the presence of various concentrations of bicarbonate in the copresence of PMA. Signal for DNA is depicted in green, and signal for citrullinated histone H3 is displayed in red. The scale bar represents 20 μm.

**Figure 6:**
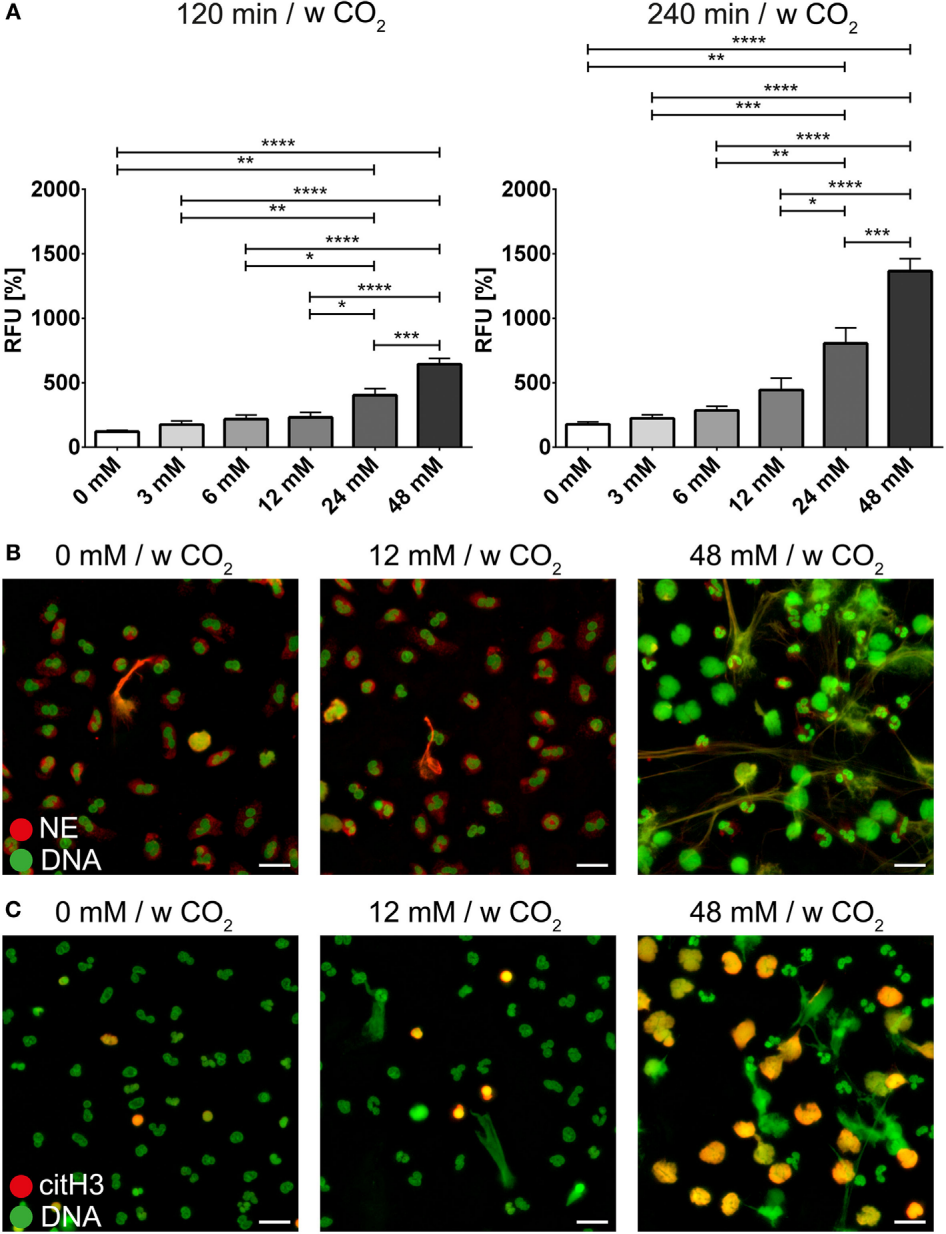
**The bicarbonate to CO_2_ ratio influences the neutrophil extracellular trap-inducing potential of ionomycin in RPMI**. RPMI was supplemented with various concentrations of bicarbonate in the presence of 5% CO_2_. Ionomycin was used at a concentration of 1 μM. **(A)** Fluorescence-based quantification of DNA externalization in response to various concentrations of bicarbonate after 120 min (left) and 240 min (right) in the presence of ionomycin. *n* = 5–11. **(B)** Immunocytochemical analysis of neutrophils incubated in the presence of various concentrations of bicarbonate in the copresence of ionomycin. Signal for DNA is depicted in green, and signal for neutrophil elastase is displayed in red. The scale bar represents 20 μm. **(C)** Immunocytochemical analysis of neutrophils incubated in the presence of various concentrations of bicarbonate in the copresence of ionomycin. Signal for DNA is depicted in green, and signal for citrullinated histone H3 is displayed in red. The scale bar represents 20 μm.

The bicarbonate/CO_2_ axis and the pH were also able to modify the effect of PMA or ionomycin on neutrophils. At low concentrations of bicarbonate, NET formation induced by PMA (Figure 5A; Video S4 in Supplementary Material) or ionomycin (Figure 6A) was significantly reduced compared to 24 and 48 mM bicarbonate, respectively. Immunocytochemistry for NE and citH3 demonstrated increased amounts of NETs positive for NE and/or citH3 in the presence of bicarbonate. Using live cell imaging, we analyzed the morphological changes in neutrophils under these stimulatory conditions and observed the markedly increased chromatin externalization and decondensation in the presence of bicarbonate. Overall, these observations were in line with the results of the quantitative fluorescence-based assay (Figures 5B and 6B; Figures S7–S10 in Supplementary Material).

**Figure 7:**
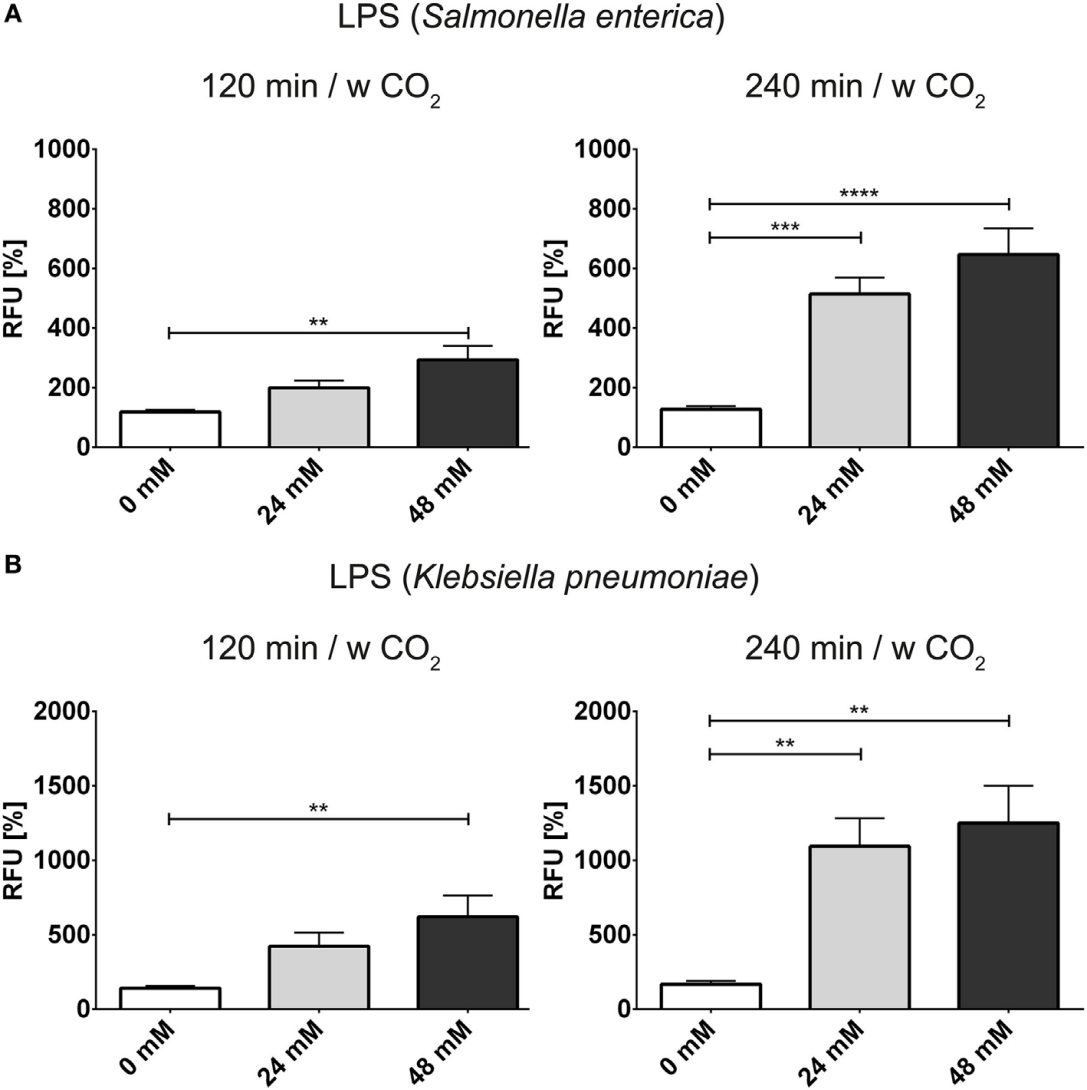
**The bicarbonate to CO_2_ ratio influences the neutrophil extracellular trap-inducing potential of LPS in RPMI**. RPMI was supplemented with various concentrations of bicarbonate in the presence of 5% CO_2_. LPS was used at a concentration of 2.5 μg/ml. **(A)** Fluorescence-based quantification of DNA externalization in response to various concentrations of bicarbonate after 120 min (left) and 240 min (right) in the presence of LPS from *Salmonella enterica*. *n* = 9 **(B)**. **(A)** Fluorescence-based quantification of DNA externalization in response to various concentrations of bicarbonate after 120 min (left) and 240 min (right) in the presence of LPS from *Klebsiella pneumoniae*. *n* = 9.

**Figure 8:**
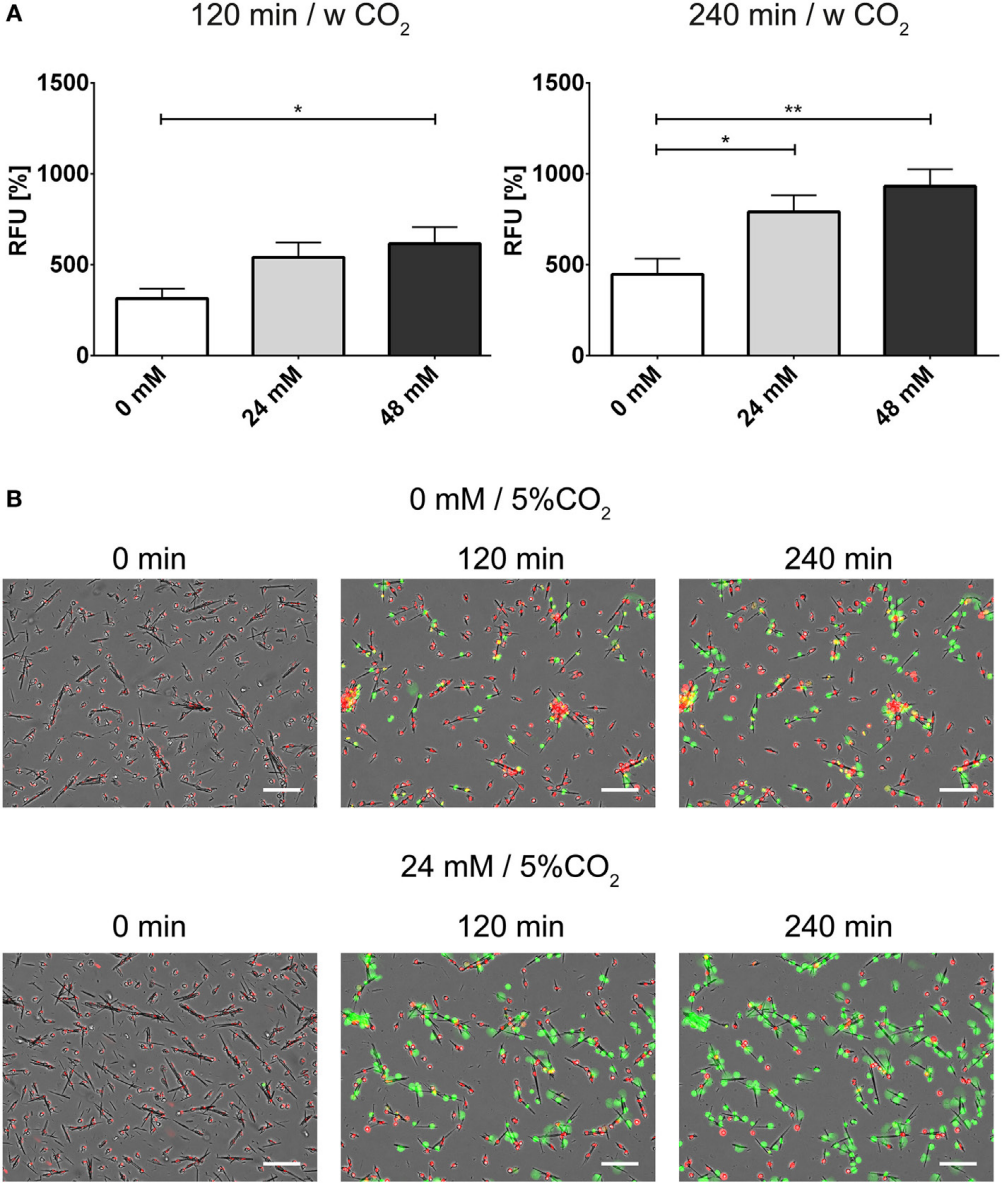
**The bicarbonate to CO_2_ ratio influences the neutrophil extracellular trap-inducing potential of monosodium urate crystals (MSU) in RPMI**. RPMI was supplemented with various concentrations of bicarbonate in the presence of 5% CO_2_. MSU was used at a concentration of 300 pg/cell. **(A)** Fluorescence-based quantification of DNA externalization in response to various concentrations of bicarbonate after 120 min (left) and 240 min (right) in the presence of MSU. *n* = 8–10. **(B)** Still movie of PMNs coincubated with MSU either under relative hypercapnia (0 mM bicarbonate/5% CO_2_) or 24 mM bicarbonate/5% CO_2_. Signal for DNA of cells with intact plasma membrane integrity is depicted in red (Hoechst 33342), and signal for extracellular DNA and inside of necrotic cells is depicted in green. The numbers indicate the time in min after addition of MSU, the scale bar represents 20 μm.

In order to further analyze the influence of bicarbonate in the media in response to potential physiological inducers of NET formation, we deployed lipopolysaccharides from *K. pneumoniae* and *S. enterica*, respectively (Figures 7A,B). LPS-stimulated NET formation was reduced under conditions of relative hypercapnia as highlighted by fluorescence-based quantification of DNA externalization (Figure 7) and immunocytochemistry (Figures S11 and S12 in Supplementary Material). As reported by Pieterse and colleagues, LPS from *S. enterica* is a poor inducer of NET formation in the absence of platelets (12). Confirming this data, we observed only a twofold increase as compared to baseline NET formation at 24 mM bicarbonate/5% CO_2_. Similarly, NET formation was also not pronounced at relative hypocapnia, further highlighting the necessity of platelets for the induction of NET formation by this particular subtype of LPS (12). In addition, we tested the influence of the bicarbonate to CO_2_ ration on the NET-inducing potential of monosodium urate crystals (MSU). NET release in response to monosodium urate was decreased at a low bicarbonate to CO_2_ ratio (Figures 8A,B; Video S6 in Supplementary Material). Taken together, our findings indicate an important role of the extracellular pH and the bicarbonate/CO_2_ axis in the signal integration of NET formation.

**Figure 9:**
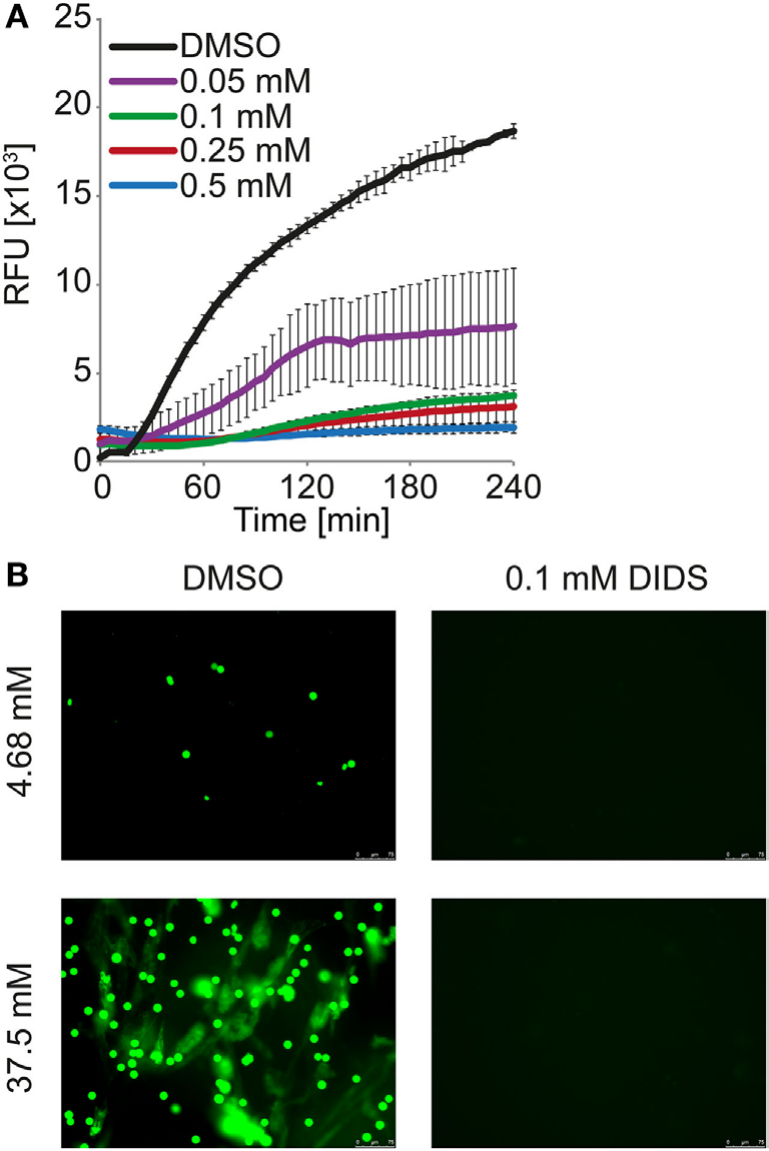
**DIDS reduces neutrophil extracellular trap formation observed at high concentrations of bicarbonate**. HBSS was supplemented with 37.5 mM bicarbonate at ambient CO_2_. **(A)** Kinetic of DNA externalization quantified in a fluorescence-based assay. DIDS was supplied at the indicated concentrations, DMSO served as vehicle control. *n* = 3. **(B)** Neutrophils were incubated in HBSS with 4.68 or 37.5 mM bicarbonate for 2 h. The media were supplemented with 0.1 mM DIDS or DMSO vehicle control. Subsequently, cells were fixed with 1% paraformaldehyde and DNA was stained with 5 μM Sytox Green (green). Images were taken using a BZ-X710 microscope.

We considered direct effects of the change in pH on phospholipid membrane integrity in neutrophils. Strikingly, the anion channel inhibitor 4,4′-diisothiocyano-2,2′-stilbenedisulfonic (DIDS) drastically reduced DNA externalization (Figure 7A). Microscopic analysis revealed that NET formation was strongly reduced/delayed by DIDS (Figure 7B; Video S5 in Supplementary Material). DIDS was also able to inhibit NET formation in response to PMA, ionomycin, and MSU at 24 mM bicarbonate/5% CO_2_ (Figure 9; Figure S13 in Supplementary Material). Since DIDS specifically inhibits anion channels, a protein-dependent mechanism is likely to be causative in pH control of NET formation. It is likely that alterations of the pH trigger a molecular program that increases the susceptibility of neutrophils to release NETs.

## Discussion

Our data indicate that the triangular relationship of CO_2_, bicarbonate and pH strongly influences the capacity of neutrophils to form NETs. We observed that NET formation is decreased in conditions with a high ratio of CO_2_ to bicarbonate even in the presence of biochemical NET-inducers. A low ratio resulted in NET formation in the absence of added inducers and augmented the NET-inducing potential of multiple triggers including PMA, ionomycin, monosodium urate, and lipopolysaccharides. This effect was observed both in HBSS and RPMI, yet markedly enhanced in the absence of extracellular amino acids as in HBSS. In the absence of CO_2_ and bicarbonate in HEPES-buffered media, the independent contribution of the pH was assessed. These studies indicate that the extracellular pH has an important influence on the capacity of neutrophils to release NETs but can only partially account for the drastic NET formation in the presence of bicarbonate/CO_2_.

Our data is in line with findings from other groups that observed that neutrophil function is depending on extracellular pH. Trevani and colleagues reported that extracellular acidification enhances specific functions of human neutrophils (13). They observed that extracellular acidosis transiently increases intracellular calcium and results in upregulation of the adhesion-mediating surface marker CD18. The stimulatory effects of conventional agonists were markedly increased by a low pH and the neutrophils responded with more production of H_2_O_2_ and increased release of myeloperoxidase (13). A more recent publication confirms these results, indicating that a pH of 6.0 prolongs neutrophil survival and increases phagocytosis of bacteria; however, phagolysosomal killing is decreased (14). Neutrophils cultivated in alkaline conditions show decreased survival compared to neutral or acidic environments (15).

Importantly, the effects of pH on immune and cellular functions are very broad and affect a multitude of cellular signaling molecules (16). Assay conditions may therefore strongly affect the specific results but may not necessarily depict the *in vivo* situation. Trevani and colleagues also highlighted the role of CO_2_ and bicarbonate to neutrophil function, since intracellular acidification in response to extracellular hydrochloric acid challenge was less pronounced in bicarbonate free medium (13, 16). The importance of the choice of the acidifying agent is highlighted by findings showing that hydrochloric acid induces an inflammatory response in stimulated RAW 264.7 cells, whereas acidification with lactate leads to an anti-inflammatory phenotype (17). However, the buffering agent is not solely responsible for the diverse effects reported through the years as indicated by inconsistent results even among studies using one and the same buffering system (13, 14, 16, 18).

We have observed a decrease of intracellular pH in conditions with a high ratio of CO_2_ to bicarbonate and an increase of intracellular pH in alkaline conditions. The change of pH in both directions was accompanied by an increase in intracellular calcium; however, elevated levels of the ion were only observed in response to alkalinization. An increase of cytosolic pH in neutrophils has been reported for a variety of stimuli, including PMA, ionomycin, and platelet-activating factor (19–21). We hypothesize that intracellular alkalinization by a high extracellular pH renders neutrophils more responsive to NET-inducing agents. Intracellular alkalinization likely triggers the same signals that follow stimulus-induced alkalinization. The concomitant increase in calcium is in line with this hypothesis, since calcium is required for several enzymes involved in NET formation.

The intracellular pH of neutrophils is thought to be mainly regulated by Na^+^/H^+^ and Cl^−^/HCO_3_^−^ antiporters. Recently a Na^+^/HCO_3_^−^ cotransporter has been identified (22, 23). Efflux of chloride is commonly accompanying activation of neutrophils (24). Substitution of chloride with glucuronate leads to an outward flux of chloride (25). A high extracellular concentration of bicarbonate is likely recapitulating a similar effect. Interestingly, the broadly acting anion channel inhibitor DIDS was able to inhibit bicarbonate-induced NET release in a time and dose-dependent manner. The altered behavior of neutrophils at different pH is of special interest in the setting of cystic fibrosis (CF). The airway mucus and intestinal and pancreatic fluid in patients with CFTR mutations are strongly altered in the pH. Additionally, alterations in neutrophil intracellular pH homeostasis have been implicated in CF (26). It is tempting to speculate that these pH changes influence NET formation observed in patients with CF (27).

**Figure 10:**
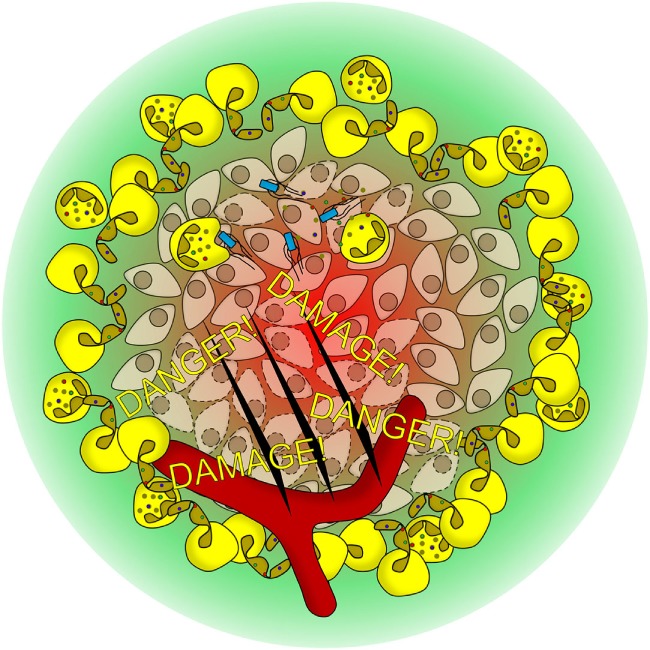
**Hypothetical model of how the pH modulates neutrophil function *in vivo***. Bacterial infection or tissue damage results in the recruitment of neutrophils. With ongoing inflammation, the pH acidifies (red to green gradient), rendering neutrophils unable to perform neutrophil extracellular trap (NET) formation. However, other antibacterial mechanism like phagocytosis or degranulation is not affected by the pH. NET formation is observed in the well-vascularized border region of the inflammation, where a neutral pH environment is found. The NETs shield off the infected area and prevent the spreading of microbes and danger-associated molecular patterns.

Our results give an interesting perspective on the role of the pH in inflammatory processes. We have observed that NET formation is substantially decreased in an acidic environment. In inflamed areas, pH values of as low as 5.5 have been reported (16). Although the observations presented here are limited to *in vitro* experiments, we will discuss possible implications for the *in vivo* situation (Figure 10).

In areas of hypoxia, such as an inflammatory focus or ischemic tissue, anaerobic glycolysis will lead to an increased formation of lactic acid and to tissue acidification. The border of the inflammatory areas is characterized by a steep increase in oxygen saturation as well as a gradient from acidic to neutral pH (Figure 10). We hypothesize that neutrophils detect the border of the inflamed area in part by this pH gradient. NET formation at elevated pH values might then primarily function as a barrier to wall-off infected or inflamed areas and to prevent invasion of the organism with pathogens or to prevent spreading of danger-associated molecular patterns from necroinflammatory areas (28). The pH might also serve as an indicator for neutrophils to sense the progress of inflammation. In the acidic center of the inflammatory focus, pathogens might still be traced and phagocytosed whereas in the periphery, the strategy is to shield the non-inflamed tissue (Figure 10). Restitution of serum pH in the setting of reperfusion may then promote NET formation and contribute to reperfusion injury.

The tumor microenvironment can technically be considered a necroinflammatory area, with hypoxic, necrotic cores, and areas of hypervasculation due to aberrant neoangiogenesis. In a similar manner, also the tumor environment is characterized by steep intratumoral and peritumoral pH gradients. Besides the presence of tumor-associated neutrophils, tumor-bearing mice exert a prethrombotic phenotype. Recent studies have causally implicated to increased NET formation as a hypercoaguable state (29–31). Moreover, NETs reportedly promote metastasis. The existence and the role of NETs in the microenvironment surrounding the tumor are far less examined. Following our hypothesis, we would expect neutrophils to undergo NET formation in contact to necrotic tumor tissue spatially influenced by the local pH. It will be the focus of future studies to determine the biological role of NETs adjacent to tumors, as these NETs could temporarily prevent expansion of the tumor or be hijacked by the malignancy to promote its spread. Such complexity is in line with recent reports about neutrophils being involved in both the initiation and resolution of inflammation (32–34).

Apart from these thoughts on the role of the pH on neutrophil function *in vivo*, our results have far-reaching technical implications for the multitude of studies which currently examine the effect of chemical interference with NET formation. Experimentators need to be aware of the *ménage-à-trois* of bicarbonate, CO_2_, and pH. Even mild changes in the ratio of CO_2_ to bicarbonate and the pH may severely impact the outcome of an experiment. An inhibitory effect of a compound *in vitro* might solely be due to pH alterations of the media. In a standard lab incubator, simply opening the door to enter a well plate profoundly changes the incubator atmosphere for up to 15 min. Therefore, using a gas-control module in a microplate reader will significantly improve the quantification of DNA externalization. pH effects are especially pronounced for ionomycin, which shows increasing binding affinity to Ca^2+^ with increasing medium pH (35). Ionomycin induces a calcium and PADI4-dependent subroutine of NET release and is therefore in the center of therapeutic research (36–39). Small variations of the extracellular pH may easily mask the effects of drugs targeting the potential of neutrophils to externalize chromatin. Likewise, the strong inter- and intra-individual variations in the formation of NETs could at least in part be related to unstable experimental settings (40).

Altogether, our findings highlight the necessity of optimized pH control in NET assays. A close attention to pH-related issues will increase the validity of experiments and allow higher reproducibility and detection of more subtle changes.

## Author Contributions

CM, AM, and ML planned and performed most of the experiments, conducted data analysis, and wrote the manuscript. SP, SG, JH, DK, and MB performed experiments and conducted data analyses. LM, CB, and GS provided scientific input and wrote the manuscript. PT and RF performed microscopy and scientific input. ML and MH supervised the project, planned and conducted experiments, analyzed data, and wrote the manuscript. All the authors read and approved the manuscript.

## Conflict of Interest Statement

The authors declare that the research was conducted in the absence of any commercial or financial relationships that could be construed as a potential conflict of interest.

## Supplementary Material

Figure S1 **Bicarbonate induces formation of neutrophil extracellular traps in HBSS (full)**. Immunocytochemical analysis of neutrophils incubated in the presence of various concentrations of bicarbonate at ambient CO_2_. Signal for DNA is depicted in green, and signal for neutrophil elastase is displayed in red. Single channel full images and overlays of the sections from Figure 1B are depicted. The scale bar represents 80 μm.

Figure S2 **Formation of neutrophil extracellular traps in HBSS is reduced at 5% CO_2_ (full)**. Immunocytochemical analysis of neutrophils incubated in HBSS containing various concentrations of bicarbonate in the presence of 5% CO_2_. Signal for DNA is depicted in green, and signal for neutrophil elastase is displayed in red. Single channel full images and overlays of the sections from Figure 1C are depicted. The scale bar represents 80 μm.

Figure S3 **Sytox Green binding to DNA is not affected by medium pH**. Calf thymus DNA was diluted to the indicated concentrations in RPMI containing various concentrations of bicarbonate and 2.5 μM Sytox Green. Fluorescence was assessed with quantitative fluorimetry similar to the detection of DNA externalization. Depicted is the mean of two independent measurements.

Figure S4 **High extracellular pH values induce formation of neutrophil extracellular traps in the absence of bicarbonate/CO_2_ in RPMI (full)**. Immunocytochemical analysis of neutrophils incubated in RPMI containing 20 mM HEPES adjusted to various pH values. Signal for DNA is depicted in green, and signal for neutrophil elastase is displayed in red. Single channel full images and overlays of the sections from Figure 3B are depicted. The scale bar represents 80 μm.

Figure S5 **Bicarbonate in RPMI induces chromatin externalization in the absence of proper CO_2_ control**. Isolated neutrophils were incubated for 30 min either at ambient CO_2_ (bottom) or in the presence of 5% CO_2_ (top). Cells were fixed with 1% paraformaldehyde for 30 min and stained with 5 μM Sytox Green and analyzed on a BZ-X710 microscope. Maximum intensity projection of Z-stacks and gamma correction were performed to increase depth of field and to allow proper display of neutrophil extracellular traps and nuclei on the same image, respectively. Post-processing of pictures was performed with Photoshop CS5. The scale bar represents 40 μm.

Figure S6 **Bicarbonate-induced formation of neutrophil extracellular traps in RPMI (full)**. Immunocytochemical analysis of neutrophils incubated in RPMI containing various concentrations of bicarbonate in the presence of 5% CO_2_. Signal for DNA is depicted in green, and signal for neutrophil elastase is displayed in red. Single channel full images and overlays of the sections from Figure 4B are depicted. The scale bar represents 80 μm.

Figure S7 **The bicarbonate to CO_2_ ratio influences the neutrophil extracellular trap-inducing potential of PMA in RPMI – neutrophil elastase (NE) staining (full)**. Immunocytochemical analysis of neutrophils incubated in RPMI containing various concentrations of bicarbonate in the presence of 5% CO_2_ and 10 ng/ml PMA. Signal for DNA is depicted in green, and signal for NE is displayed in red. Single channel full images and overlays of the sections from Figure 5B are depicted. The scale bar represents 80 μm.

Figure S8 **The bicarbonate to CO_2_ ratio influences the neutrophil extracellular trap-inducing potential of PMA in RPMI – citrullinated histone H3 (citH3) staining (full)**. Immunocytochemical analysis of neutrophils incubated in RPMI containing various concentrations of bicarbonate in the presence of 5% CO_2_ and 10 ng/ml PMA. Signal for DNA is depicted in green, and citH3 is displayed in red. Single channel full images and overlays of the sections from Figure 5C are depicted. The scale bar represents 80 μm.

Figure S9 **The bicarbonate to CO_2_ ratio influences the neutrophil extracellular trap-inducing potential of ionomycin in RPMI – neutrophil elastase (NE) staining (full)**. Immunocytochemical analysis of neutrophils incubated in RPMI containing various concentrations of bicarbonate in the presence of 5% CO_2_ and 1 μg/ml ionomycin. Signal for DNA is depicted in green, and signal for NE is displayed in red. Single channel full images and overlays of the sections from Figure 6B are depicted. The scale bar represents 80 μm.

Figure S10 **The bicarbonate to CO_2_ ratio influences the neutrophil extracellular trap-inducing potential of ionomycin in RPMI – citrullinated histone H3 (citH3) staining (full)**. Immunocytochemical analysis of neutrophils incubated in RPMI containing various concentrations of bicarbonate in the presence of 5% CO_2_ and 1 μg/ml ionomycin. Signal for DNA is depicted in green, and signal for citH3 is displayed in red. Single channel full images and overlays of the sections from Figure 6C are depicted. The scale bar represents 80 μm.

Figure S11 **The bicarbonate to CO_2_ ratio influences the neutrophil extracellular trap-inducing potential of LPS from *Salmonella enterica* in RPMI – neutrophil elastase (NE) staining**. Immunocytochemical analysis of neutrophils incubated in RPMI containing various concentrations of bicarbonate in the presence of 5% CO_2_ and 2.5 μg/ml LPS. Signal for DNA is depicted in green, and signal for NE is displayed in red. The scale bar represents 80 μm.

Figure S12 **The bicarbonate to CO_2_ ratio influences the neutrophil extracellular trap-inducing potential of LPS from *Klebsiella pneumoniae* in RPMI – neutrophil elastase (NE) staining**. Immunocytochemical analysis of neutrophils incubated in RPMI containing various concentrations of bicarbonate in the presence of 5% CO_2_ and 2.5 μg/ml LPS. Signal for DNA is depicted in green, and signal for NE is displayed in red. The scale bar represents 80 μm.

Figure S13 **DIDS interferes with neutrophil extracellular trap formation**. Upper left: fluorescence-based quantification of DNA externalization in response to 100 ng/ml PMA either in the presence (PMA + DIDS) or absence (PMA) of 0.1 mM DIDS. Unstimulated (US) controls and DIDS without inducer of neutrophil extracellular trap (NET) formation (US + DIDS) are included additionally. *n* = 3. Upper right: fluorescence-based quantification of DNA externalization in response to 1 μg/ml ionomycin either in the presence (IONO + DIDS) or absence (IONO) of 0.1 mM DIDS. US controls and DIDS without inducer of NET formation (US + DIDS) are included additionally. *n* = 3. Lower left: fluorescence-based quantification of DNA externalization in response to 300 pg/cell monosodium urate crystals (MSU) either in the presence (MSU + DIDS) or absence (MSU) of 0.1 mM DIDS. US controls and DIDS without inducer of NET formation (US + DIDS) are included additionally. *n* = 3. Depicted is the mean ± SD.

Video S1 **Bicarbonate induces formation of neutrophil extracellular traps in HBSS**. Neutrophils were cultivated in isotonic HBSS medium containing 75 mM of bicarbonate and 1% of BSA. Cells were stained with 0.1 μg/ml Hoechst 33342 (green) and 500 ng/ml propidium iodide (red). Images were captured every 30 s for 30 min. Microscopy was performed on an Axio Observer.Z1 microscope using a time-lapse shooting sequence. Time is indicated in seconds and the scale bar represents 20 μm.

Video S2 **High extracellular pH values induce formation of neutrophil extracellular traps in the absence of bicarbonate/CO_2_ in RPMI**. Neutrophils were cultivated in RPMI containing 20 mM HEPES. pH was adjusted either to pH 6.6 (top) or pH 7.8 (bottom). Cells were stained with 5 μM Sytox Green (green). Images were captured every 10 min for 240 min. Microscopy was performed on a BZ-X710 microscope using a time-lapse shooting sequence. Time is indicated in minutes and the scale bar represents 20 μm.

Video S3 **The bicarbonate to CO_2_ ratio influences formation of neutrophil extracellular traps in RPMI**. Neutrophils were cultivated in RPMI containing either 0 mM (top) or 48 mM (bottom) bicarbonate. Medium was supplied with 5% CO_2_. Cells were stained with 5 μM Sytox Green (green). Images were captured every 10 min for 240 min. Microscopy was performed on a BZ-X710 microscope using a time-lapse shooting sequence. Time is indicated in minutes and the scale bar represents 20 μm.

Video S4 **The bicarbonate to CO_2_ ratio influences the neutrophil extracellular trap-inducing potential of PMA in RPMI**. Neutrophils were cultivated in RPMI containing either 0 mM (top) or 48 mM (bottom) bicarbonate in the copresence of 10 ng/ml PMA. Medium was supplied with 5% CO_2_. Cells were stained with 5 μM Sytox Green (green). Images were captured every 10 min for 240 min. Microscopy was performed on a BZ-X710 microscope using a time-lapse shooting sequence. Time is indicated in minutes and the scale bar represents 20 μm.

Video S5 **Bicarbonate-induced formation of neutrophil extracellular traps is delayed by DIDS**. Neutrophils were cultivated in isotonic HBSS medium containing 75 mM of bicarbonate and 1% of BSA either in the absence (top) or presence (bottom) of 0.1 mM DIDS. Cells were stained with 0.1 μg/ml Hoechst 33342 (green) and 500 ng/ml propidium iodide (red). Images were captured every 30 s for 30 min. Microscopy was performed on an Axio Observer.Z1 microscope using a time-lapse shooting sequence. Time is indicated in seconds and the scale bar represents 20 μm.

Video S6 **The bicarbonate to CO_2_ ratio influences the neutrophil extracellular trap-inducing potential of monosodium urate crystals (MSU) in RPMI**. Neutrophils were cultivated in RPMI containing either 0 mM (top) or 24 mM (bottom) bicarbonate in the copresence of 300 pg/ml MSU. Medium was supplied with 5% CO_2_. Cells were stained with 5 μM Sytox Green (green) and 0.05 μg/ml Hoechst 33342 (red). Hoechst 33342 (red) stains nuclei of cells with intact plasma membrane, whereas Sytox Green (green) only stains the nuclei of necrotic cells or extracellular DNA. Images were captured every 10 min for 240 min. Microscopy was performed on a BZ-X710 microscope using a time-lapse shooting sequence. Time is indicated in minutes and the scale bar represents 20 μm.
