# Supplementary figures and images for: Ménage-à-Trois: The Ratio of Bicarbonate to CO2 and the pH Regulate the Capacity of Neutrophils to Form NETs
Source: Front Immunol. 2016 Dec 9;7:583. doi: 10.3389/fimmu.2016.00583 (PMC5145884; doi:10.3389/fimmu.2016.00583)

## Slide 1
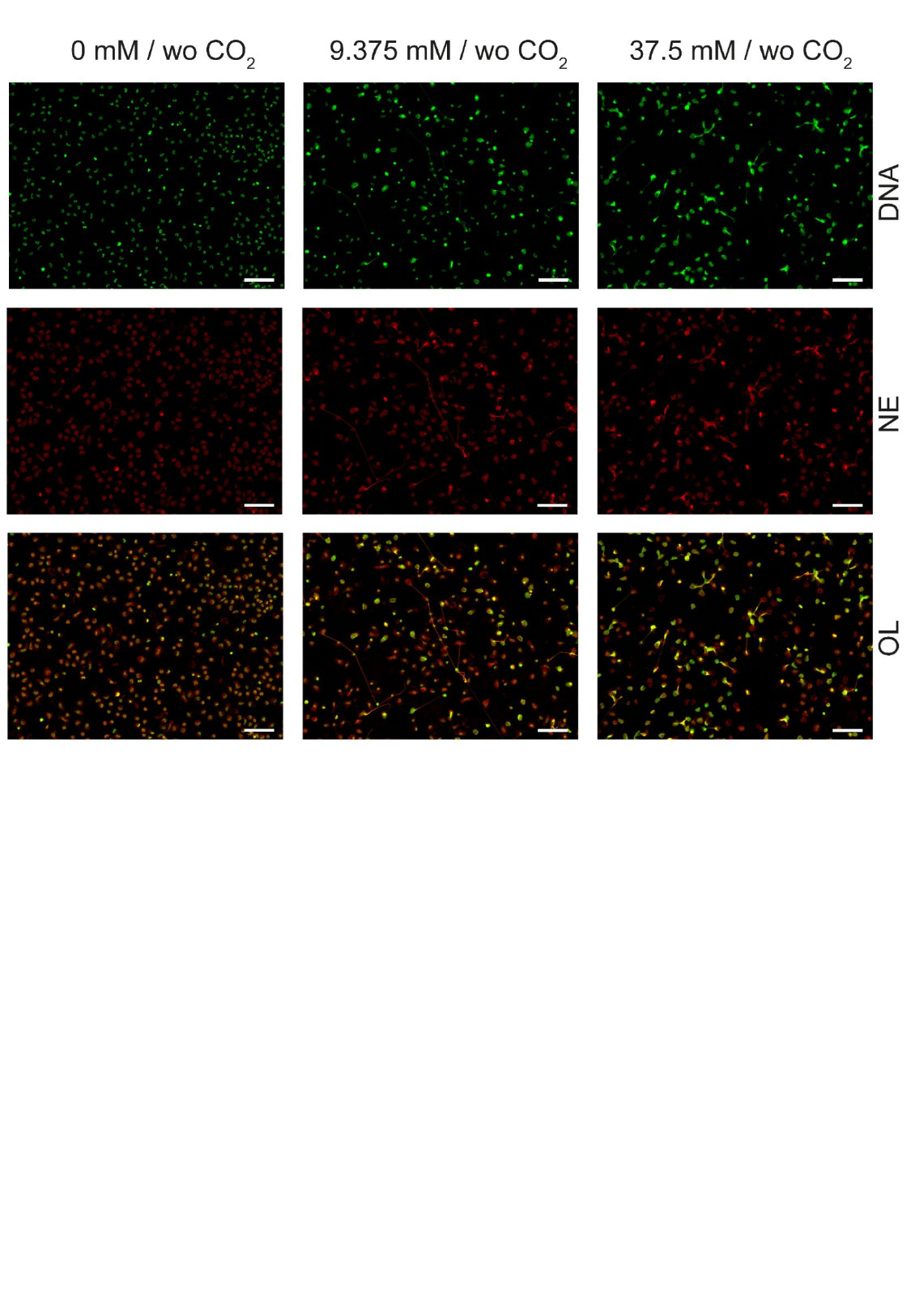

## Slide 2
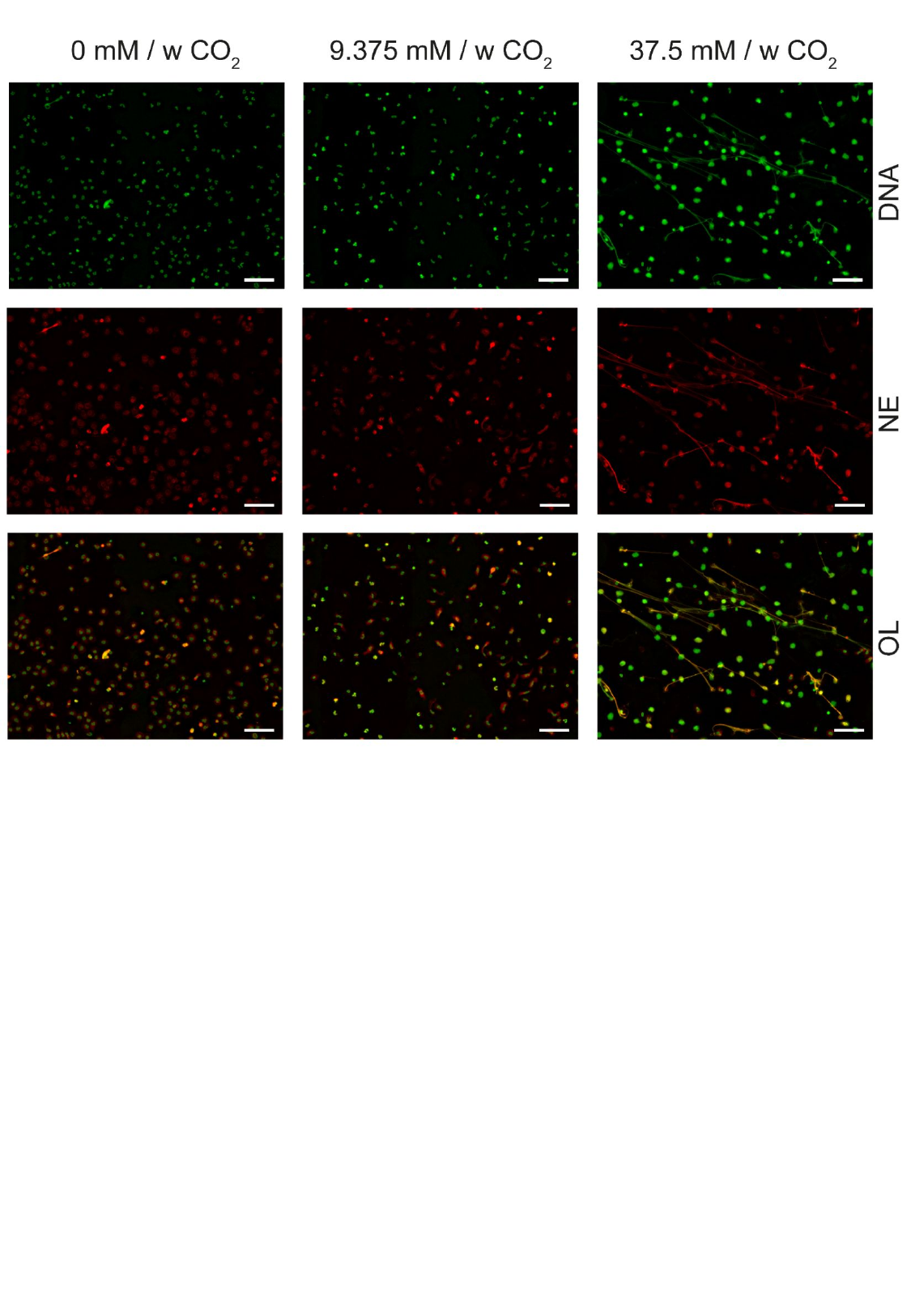

## Slide 3
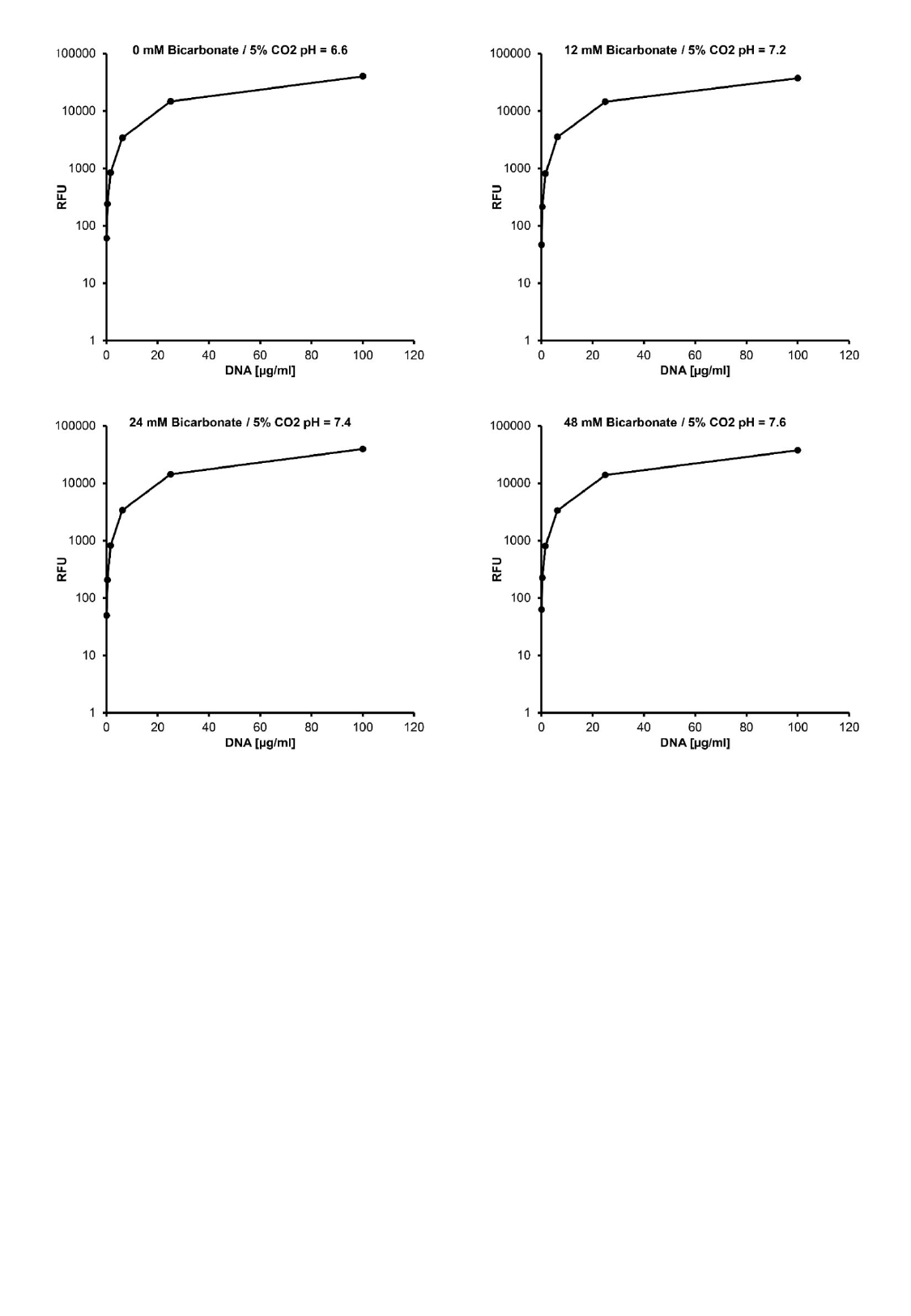

## Slide 4
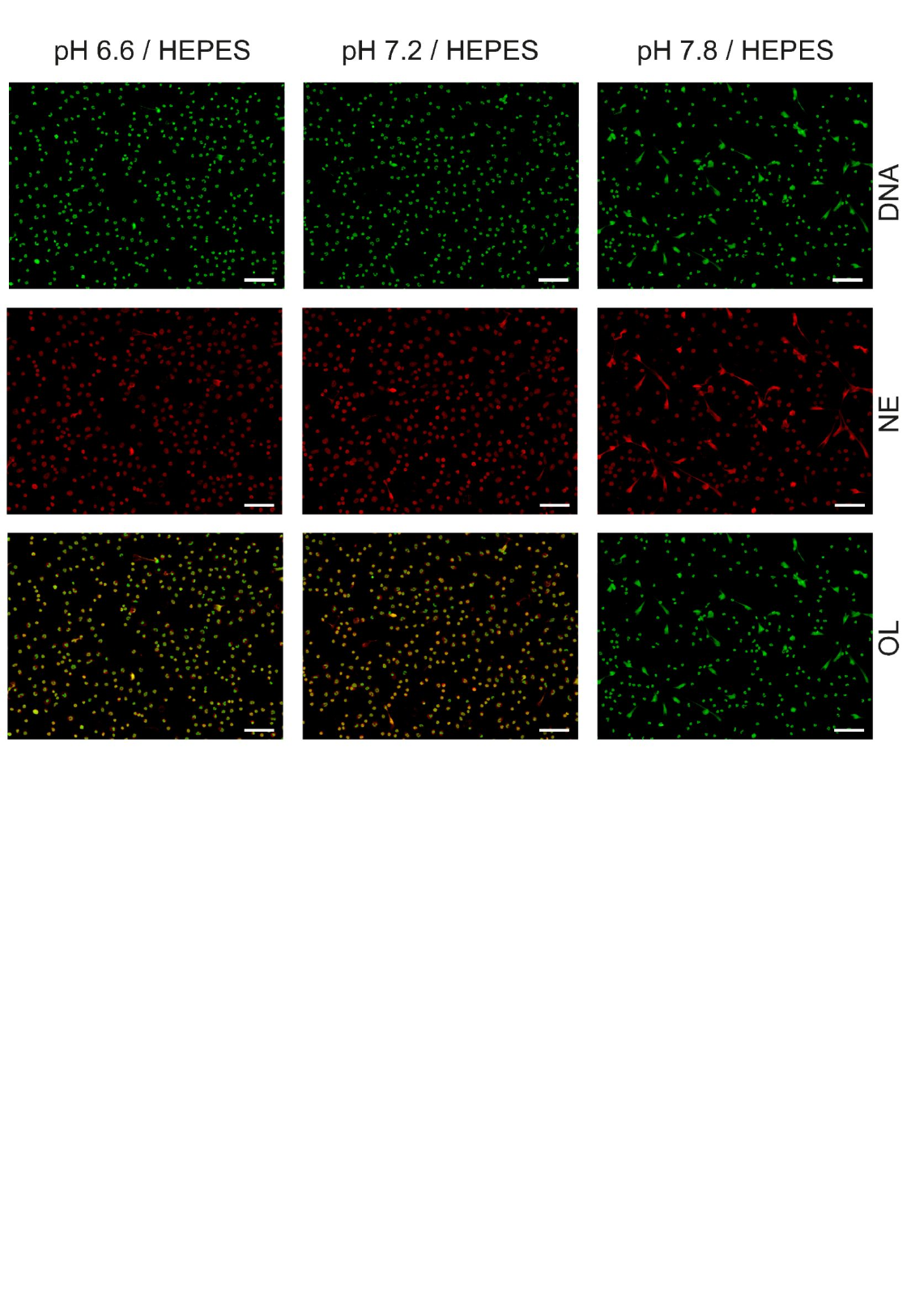

## Slide 5
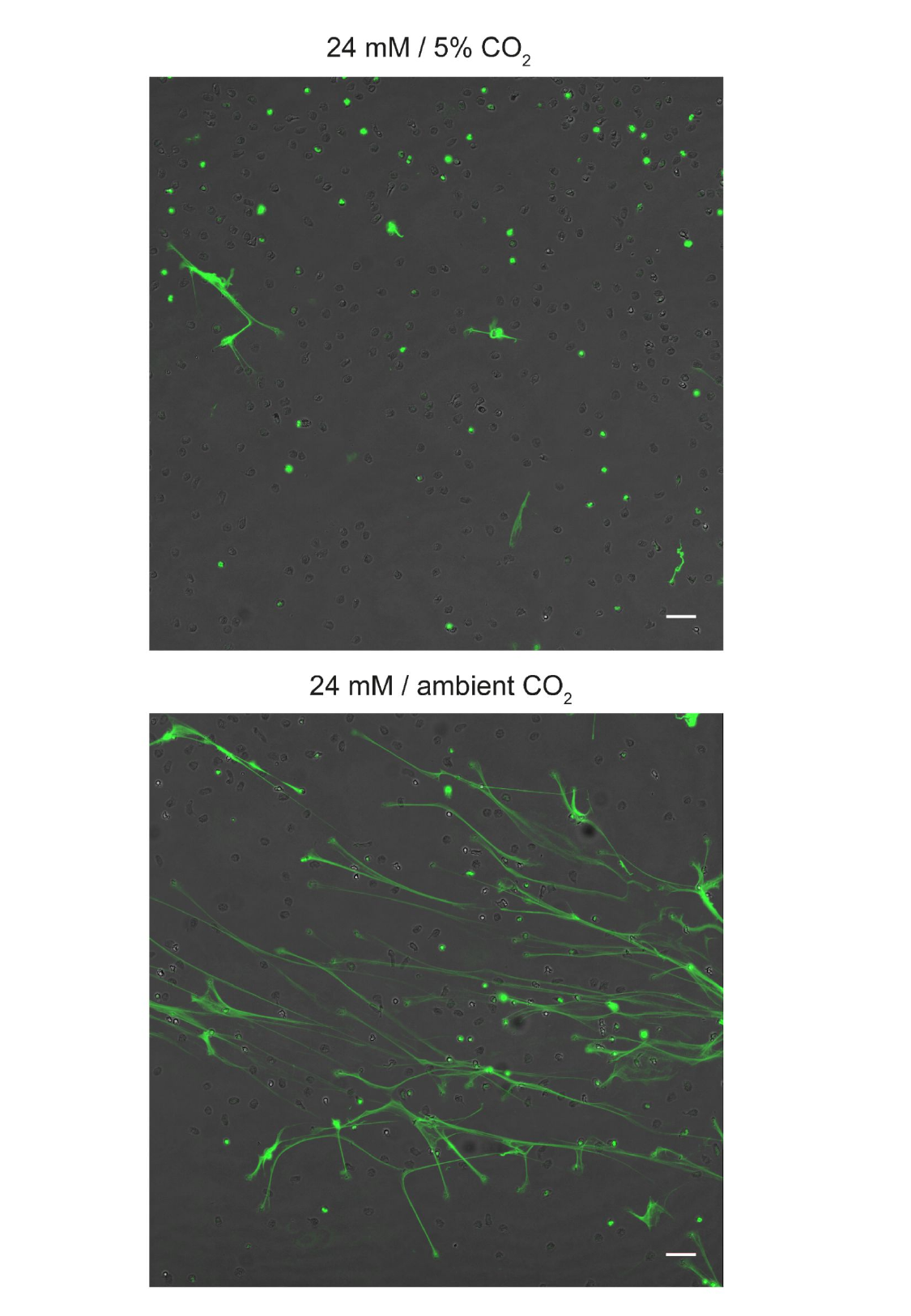

## Slide 6
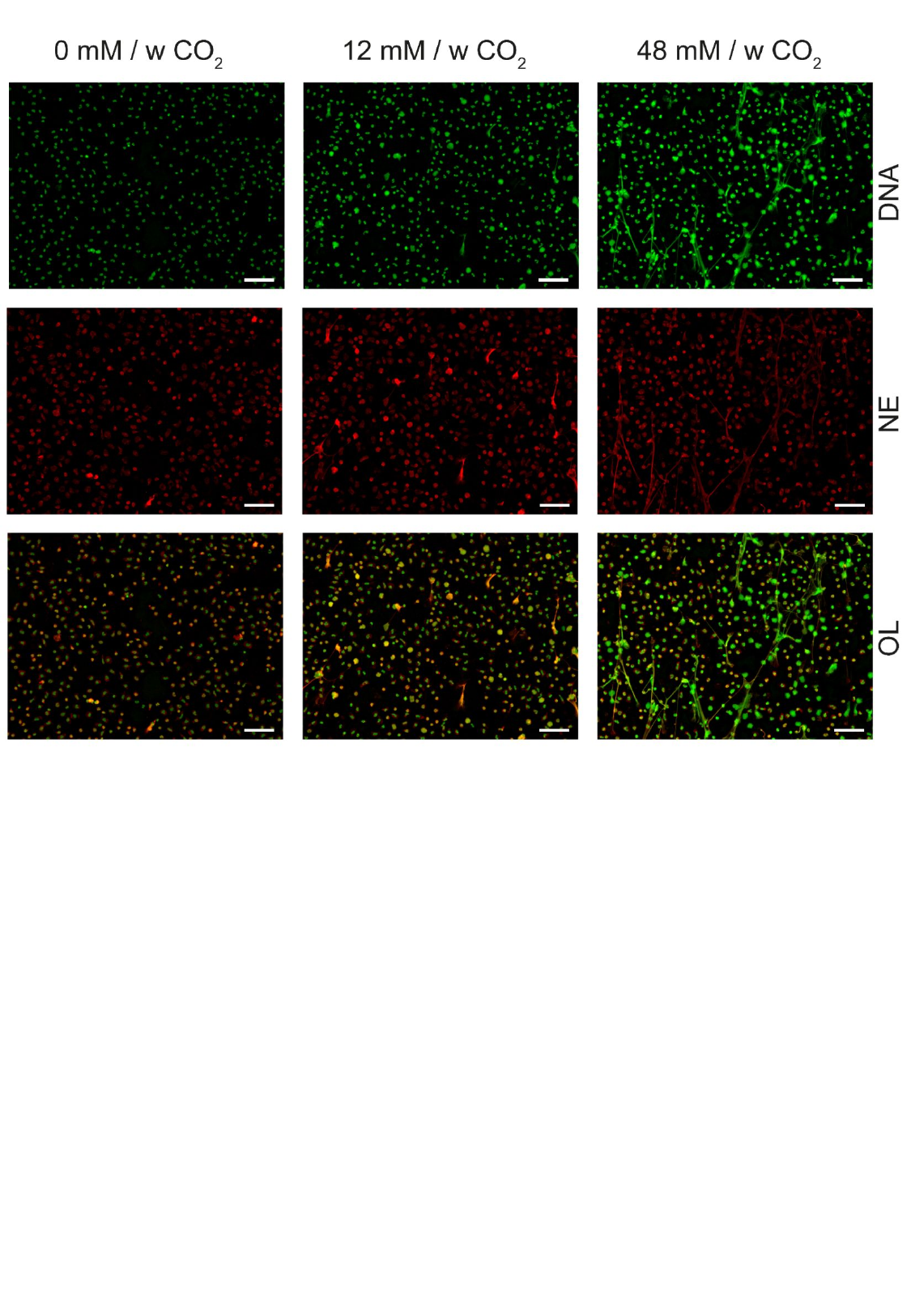

## Slide 7
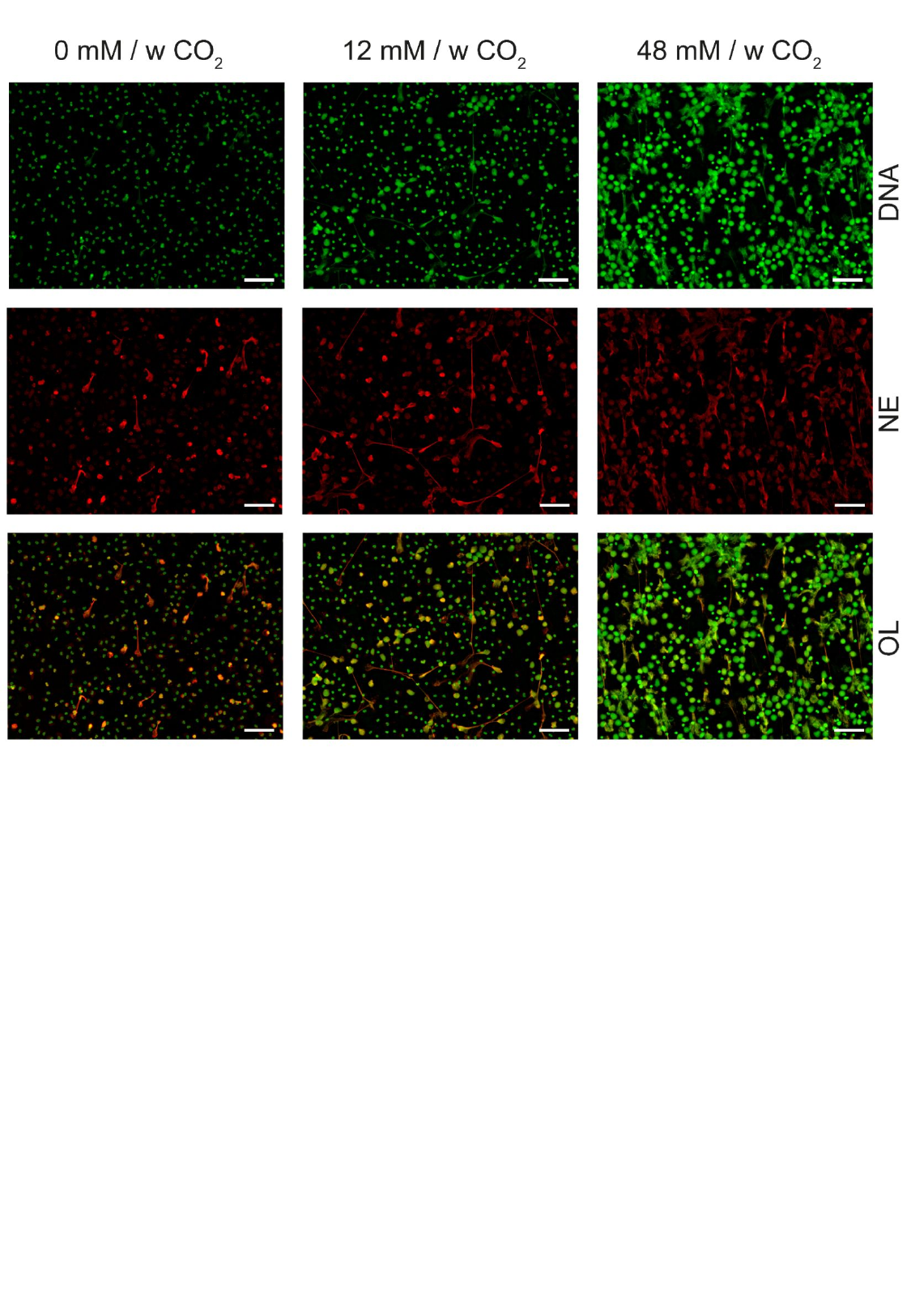

## Slide 8
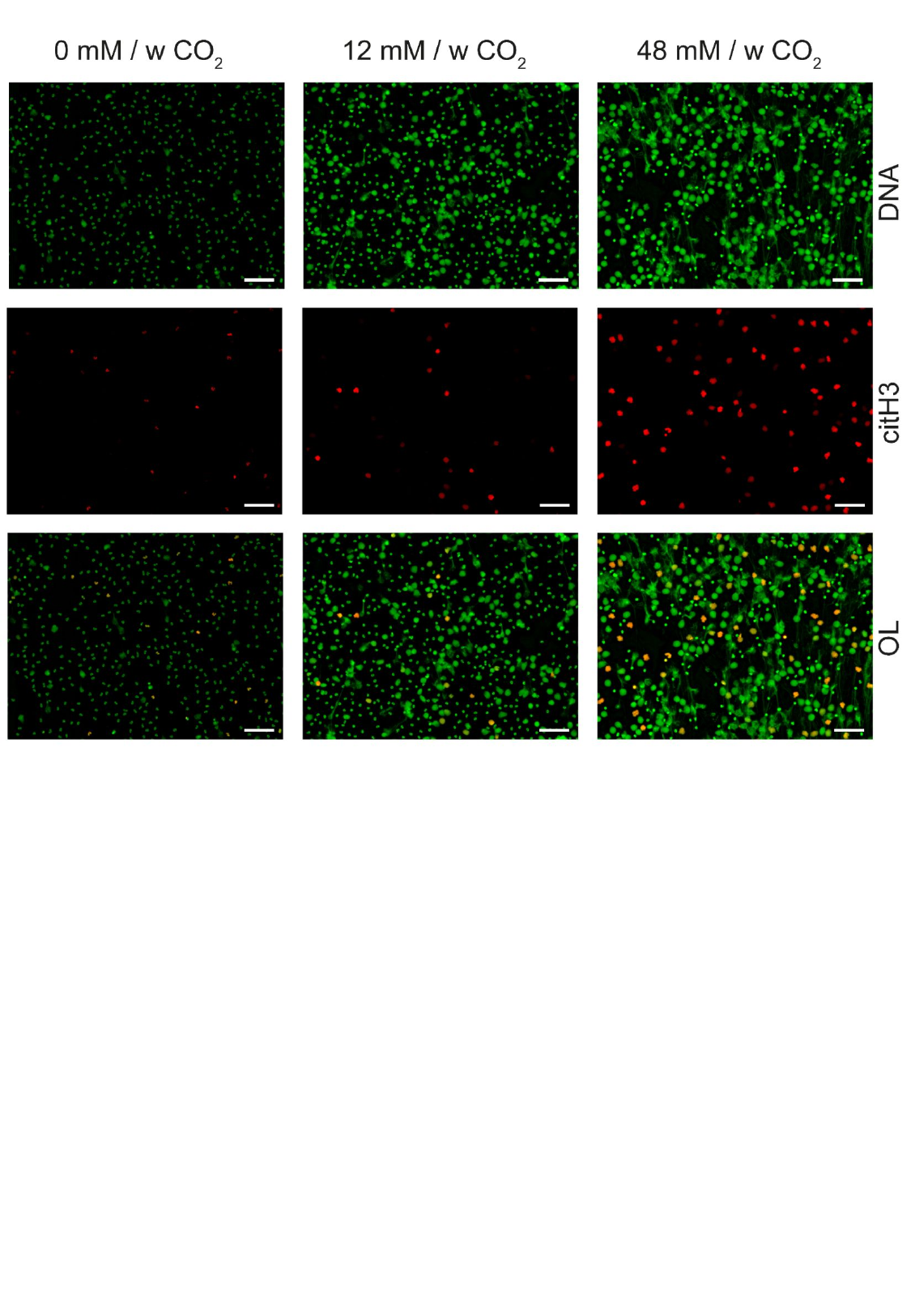

## Slide 9
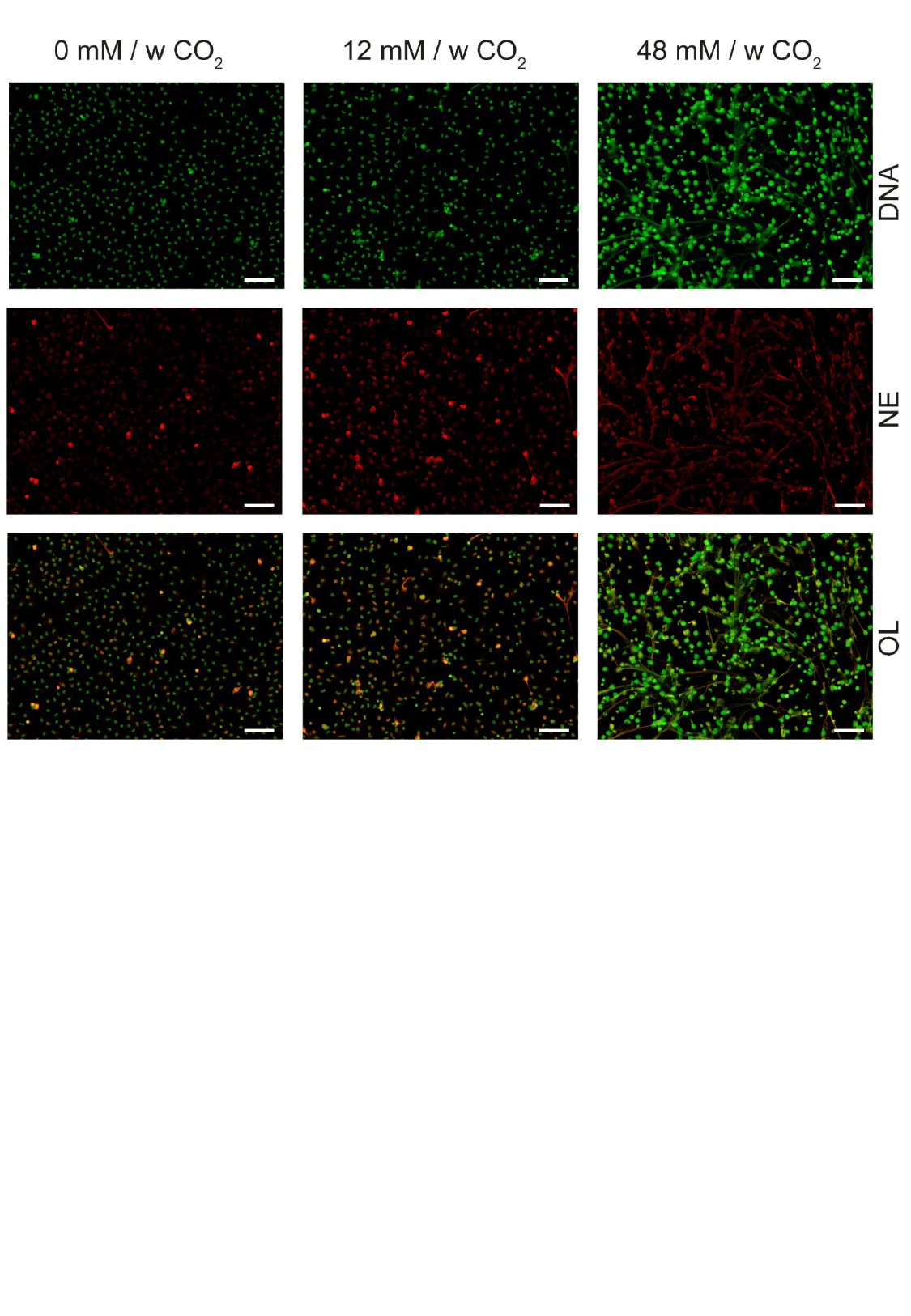

## Slide 10
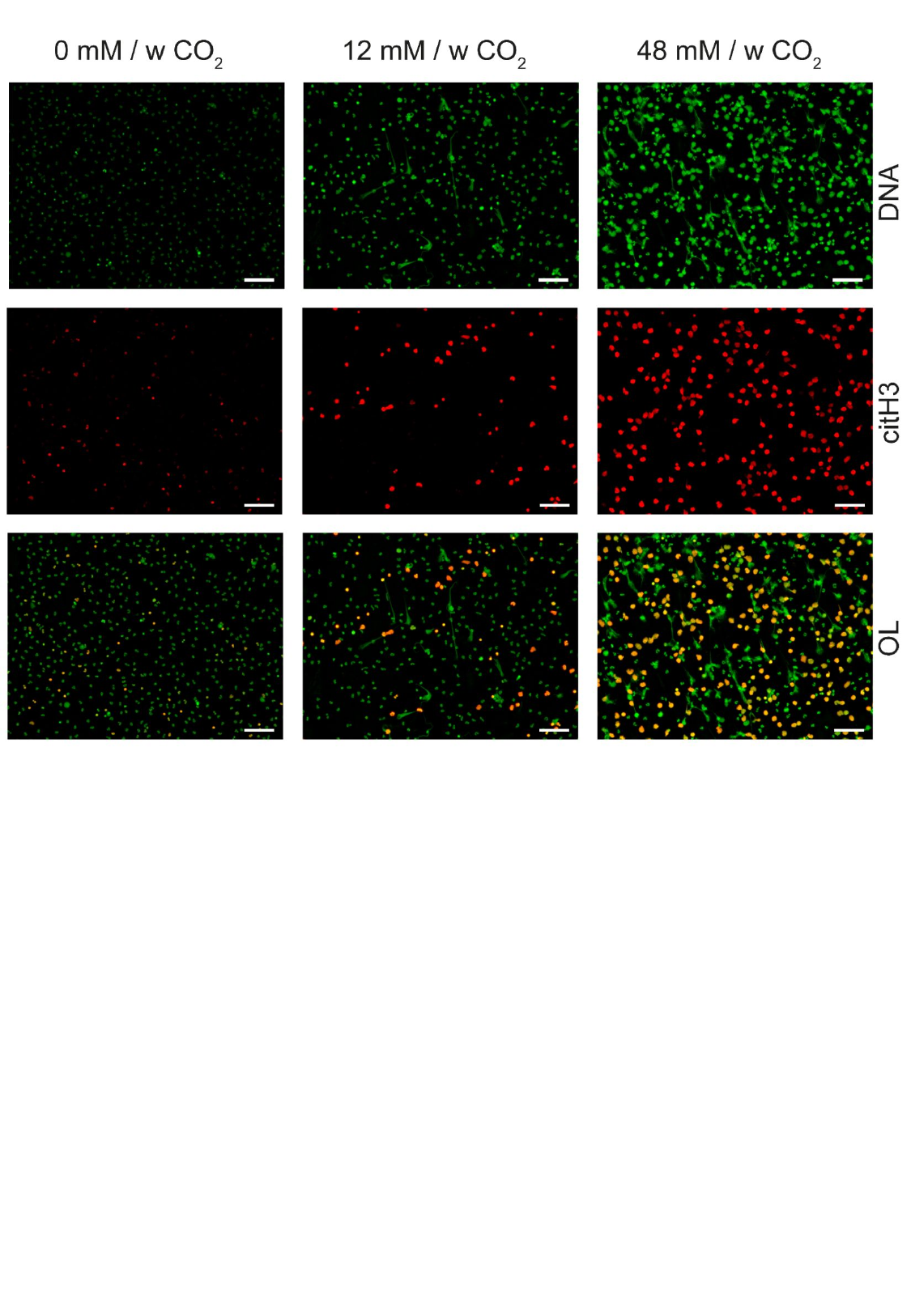

## Slide 11
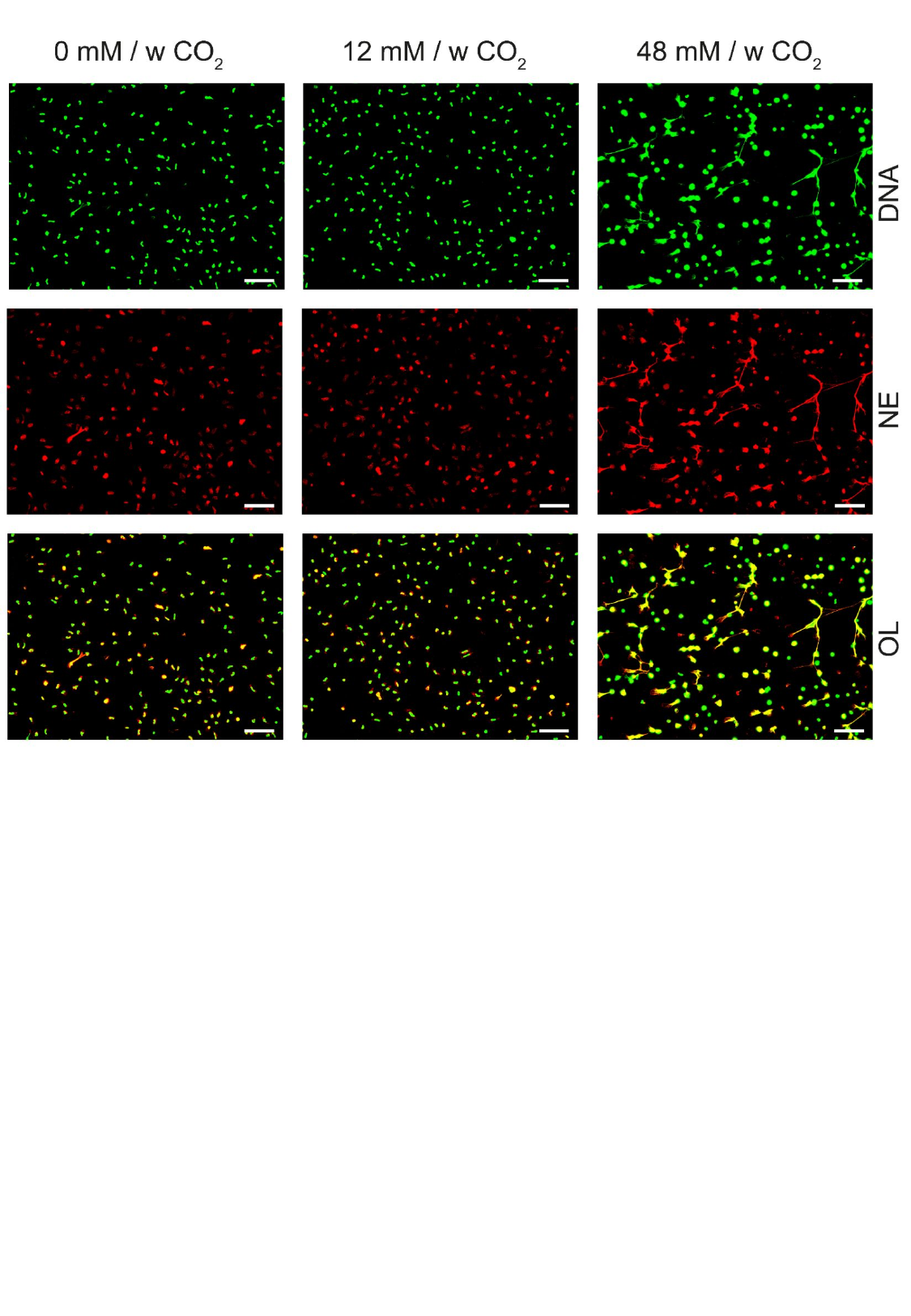

## Slide 12
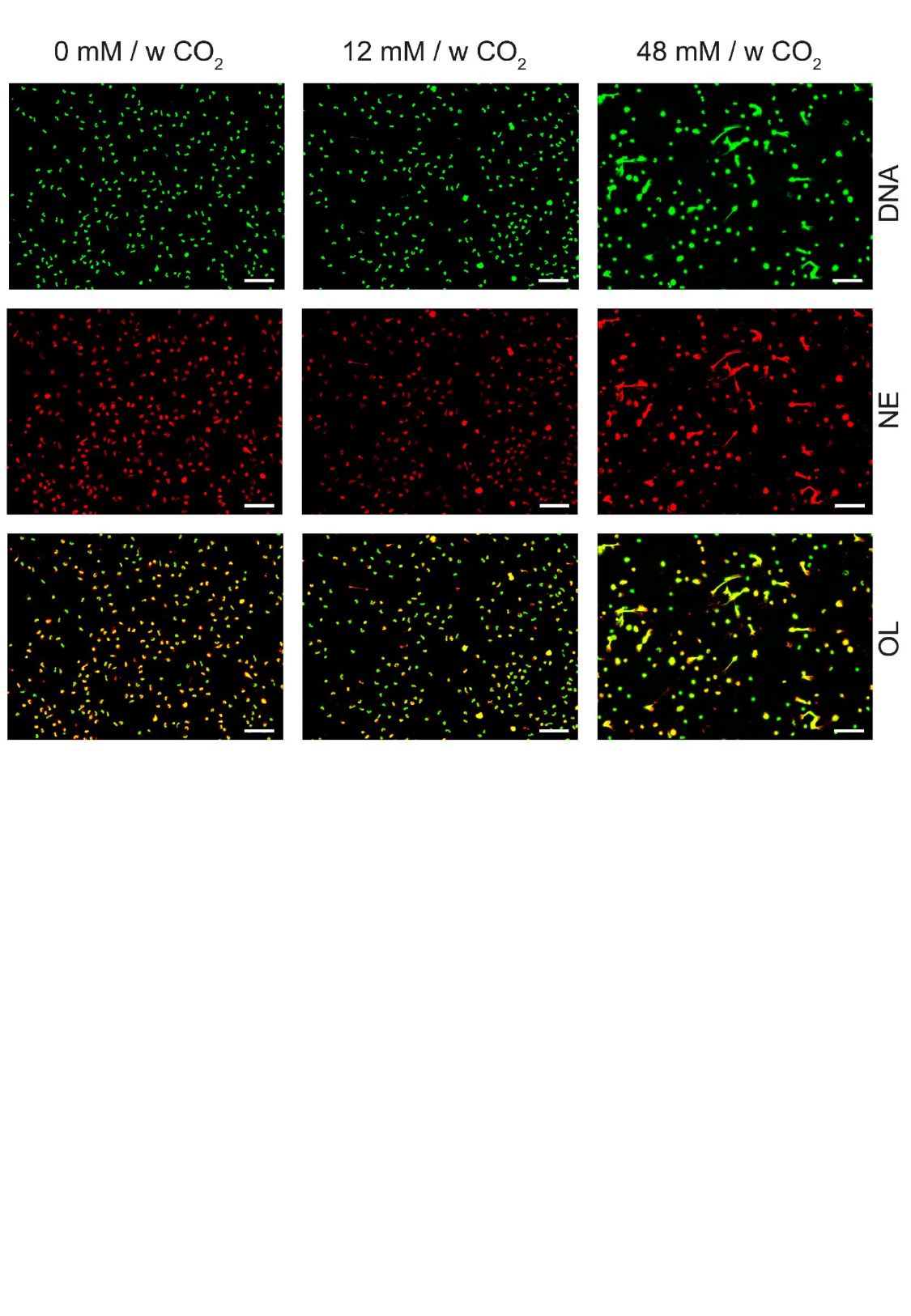

## Slide 13
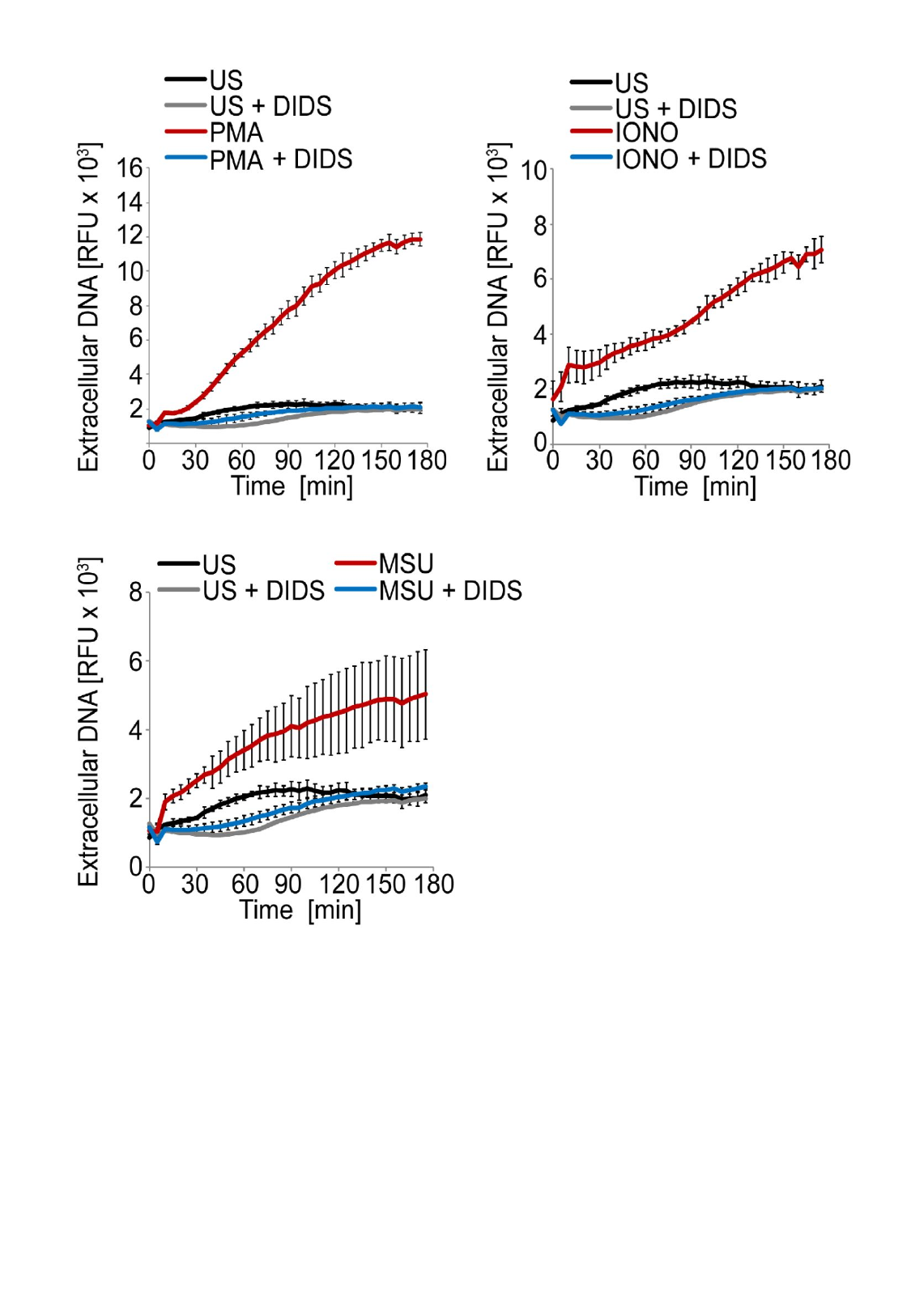

Supplement: Figure S1 — Bicarbonate induces formation of neutrophil extracellular traps in HBSS (full). Immunocytochemical analysis of neutrophils incubated in the presence of various concentrations of bicarbonate at ambient CO2. Signal for DNA is depicted in green, and signal for neutrophil elastase is displayed in red. Single channel full images and overlays of the sections from Figure 1B are depicted. The scale bar represents 80 μm. [file Presentation_1.PPTX]
